# Organ-specific features of human kidney lymphatics are disrupted in chronic transplant rejection

**DOI:** 10.1172/JCI168962

**Published:** 2025-07-15

**Authors:** Daniyal J. Jafree, Benjamin J. Stewart, Karen L. Price, Maria Kolatsi-Joannou, Camille Laroche, Barian Mohidin, Benjamin Davis, Hannah Mitchell, Lauren G Russell, Lucía Marinas del Rey, Chun Jing Wang, William J Mason, Byung Il Lee, Lauren Heptinstall, Ayshwarya Subramanian, Gideon Pomeranz, Dale Moulding, Laura Wilson, Tahmina Wickenden, Saif N. Malik, Natalie Holroyd, Claire L. Walsh, Jennifer C. Chandler, Kevin X. Cao, Paul J.D. Winyard, Adrian S. Woolf, Marc Aurel Busche, Simon Walker-Samuel, Lucy S.K. Walker, Tessa Crompton, Peter J. Scambler, Reza Motallebzadeh, Menna R. Clatworthy, David A. Long

**Affiliations:** 1 Developmental Biology & Cancer Research & Teaching Department, University College London (UCL) Great Ormond Street Institute of Child Health,; 2 UCL Centre for Kidney & Bladder Health, and; 3 UCL MB/PhD Programme, UCL, London, United Kingdom.; 4 Wellcome Sanger Institute, Wellcome Genome Campus, Hinxton, Cambridge, United Kingdom.; 5 Molecular Immunity Unit, University of Cambridge, Cambridge, United Kingdom.; 6 Central Laser Facility, Science and Technologies Facilities Council, UK Research and Innovation, Didcot, Oxfordshire, United Kingdom.; 7 Mathematical Sciences Research Centre, Queen’s University Belfast, Belfast, United Kingdom.; 8 Research Department of Surgical Biotechnology, Division of Surgery and Interventional Science,; 9 UCL Institute of Immunity and Transplantation, and; 10 UK Dementia Research Institute at UCL, London, United Kingdom.; 11 UCL Department of Pathology, Royal Free Hospital, London, United Kingdom.; 12 Department of Molecular Biology and Genetics, College of Arts and Science, Cornell University, Ithaca, New York, USA.; 13 UCL Centre for Advanced Biomedical Imaging, London, United Kingdom.; 14 School of Biological Sciences, Faculty of Biology Medicine and Health, University of Manchester, Manchester, United Kingdom.; 15 Infection, Immunity and Inflammation Research and Teaching Department, UCL Great Ormond Street Institute of Child Health, UCL, London, United Kingdom.

**Keywords:** Immunology, Nephrology, Vascular biology, Adaptive immunity, Lymph, Organ transplantation

## Abstract

Lymphatic vessels maintain tissue fluid homeostasis and modulate inflammation, yet their spatial organization and molecular identity in the healthy human kidney, and how these change during chronic transplant rejection, remain poorly defined. Here, we show that lymphatic capillaries initiate adjacent to cortical kidney tubules and lack smooth muscle coverage. These vessels exhibit an organ-specific molecular signature, enriched for *CCL14*, *DNASE1L3*, and *MDK*, with limited expression of canonical immune-trafficking markers found in other organ lymphatics, such as *LYVE1* and *CXCL8*. In allografts with chronic mixed rejection, lymphatics become disorganized and infiltrate the medulla, with their endothelial junctions remodeling from a button-like to a continuous, zipper-like, architecture. Lymphatics in rejecting kidneys localize around and interconnect tertiary lymphoid structures at different maturation stages, with altered intralymphatic and perilymphatic CD4^+^ T cell distribution. The infiltrating T cells express IFN-γ, which upregulates coinhibitory ligands in lymphatic endothelial cells, including PVR and LGALS9. Simultaneously, lymphatics acquire HLA class II expression and exhibit C4d deposition, consistent with alloantibody binding and complement activation. Together, these findings define the spatial and molecular features of human kidney lymphatics, revealing tolerogenic reprogramming accompanied by structural perturbations during chronic transplant rejection.

## Abstract 2

Jafree *et al.* combine 3D imaging and single-cell genomics, uncovering how kidney lymphatics are uniquely organised, and how they are rewired in chronic transplant rejection.

## Introduction

Lymphatics are blind-ended vessels lined by lymphatic endothelial cells (LECs); they are responsible for clearing fluid and macromolecules from the microenvironment and play a critical role in maintaining tissue homeostasis (1–3). During inflammation, lymphatics expand through lymphangiogenesis to facilitate leukocyte efflux. While their role and therapeutic potential in lymphedema (4), cardiovascular disease (5–7), cancer (8), and neuropathology or neuroinflammation (9, 10) are becoming increasingly recognized, these functions rely on organ-specific structural and molecular specialization. In the kidney, lymphatic vasculature is considered an important entity in physiology and disease (11–14). Epithelial nephrons and their associated blood capillary networks (15) underlie plasma ultrafiltration, fluid homeostasis, and acid-base balance. In contrast, although lymphatics appear in the human fetal kidney by the end of the first trimester (16), their precise spatial organization, molecular identity, and cellular interactions remain poorly defined.

Kidney transplantation, the most common solid organ transplant, has excellent short-term outcomes but is limited long-term by chronic rejection, a major cause of late allograft failure (17, 18). Chronic rejection, driven by both T cell–mediated injury and/or donor-specific antibodies targeting HLA, results in microvascular injury, interstitial fibrosis, and tubular atrophy (19, 20). Lymphatics serve as a route for trafficking of antigens and leukocytes, but their role in transplant immunity remains controversial (11, 21, 22). Lymphangiogenesis in kidney transplants (23–26) has been associated with promoting resolution of inflammation and improving allograft function (27–29) but also with alloantigen presentation and fibrosis (30, 31). These contrasting findings underscore the need to better understand lymphatic remodeling in transplant rejection and how it diverges from other kidney pathologies featuring lymphangiogenesis (32–34).

Here, we used 3D microscopy of optically cleared and immunolabeled tissue, in addition to single-cell RNA-Seq (scRNA-Seq), to map the spatial organization and molecular profile of lymphatics in the healthy human kidney. We identified blind-ended lymphatic capillaries around cortical nephron segments, with a distinct molecular signature compared with LECs from other organs. In allografts with chronic mixed rejection, lymphatic vessels expanded into the medulla and were structurally disorganized, with disrupted LEC-cell junctions. In this setting, lymphatics surrounded tertiary lymphoid structures, with altered intralymphatic and perilymphatic CD4^+^ T cell distribution. However, our molecular analyses suggest that LECs are tolerogenic and respond to T cell–derived IFN-γ by upregulating immune-inhibitory molecules. Critically, lymphatics in rejecting allografts also expressed HLA class II and exhibited complement 4d (C4d) deposition, indicative of alloantibody binding (35). Thus, our findings revealed that kidney lymphatics in chronic rejection adopt potentially compensatory tolerogenic changes but are concurrently structurally perturbed, better defining their contributions to alloimmunity.

## Results

### Characterization of lymphatic architecture and spatial relationships in the healthy human kidney.

**Figure 1:**
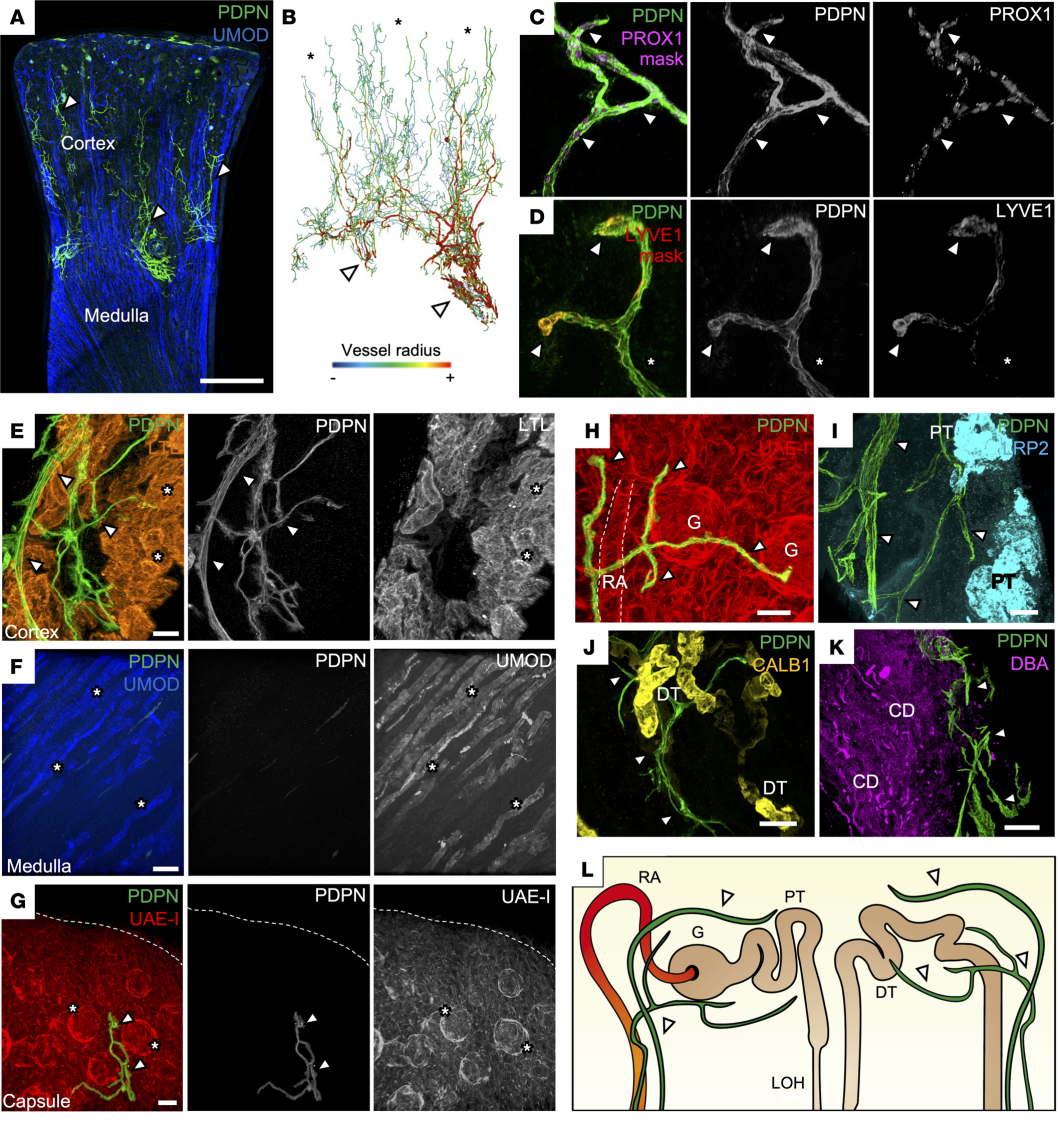
3D imaging of lymphatics and their spatial relationships in the human kidney. (**A**) Representative maximum intensity z-projection, from low-resolution confocal tile scans, of *n =* 3 human kidney tissues labeled for PDPN and UMOD, demonstrating PDPN^+^ lymphatics (arrowheads). Scale bar: 2,000 μm. (**B**) Segmented and rendered light-sheet imaging of lymphatics from the same kidney tissue in **A**, representative of *n =* 3 images. 3D color renderings represent vessel branch radii, with blue representing the smallest radius (<3.5 μm, asterisks) and red representing the largest radii (>50 μm, arrowheads). (**C** and **D**) Representative 3D reconstruction of cortical regions from *n =* 2 human kidney tissues labeled for PDPN and either PROX1 or LYVE1. The PROX1 signal and LYVE1 signal are masked to only include expression from within the vessel, demonstrating expression of PDPN^+^ cells. Sparse membrane localization of LYVE1 is demonstrated (arrowheads). Representative of 5 regions of interest imaged. Scale bars: 30 μm. (**E**–**G**) Regional localization of lymphatics (arrowheads) in the human kidney using LTL (cortex), UMOD (medulla), and UAE-I (with dotted lined delineating the capsule). Regional structures are indicated with asterisks, including proximal tubules in **E**, loops of Henle in **F**, and glomeruli in **G**. Scale bars: 70 μm (**E**), 150 μm (**F**), 100 μm (**G**). (**H**–**K**) Spatial relationships of lymphatics (arrowheads) relative to UAE-I^+^ renal arterioles (RA) and glomeruli (G) in **H**, LRP2^+^ proximal tubules (PT) in **I**, CALB1^+^ distal nephron tubules (DT) in **J**, and DBA^+^ collecting ducts (CD) in **K**. Scale bars: 50 μm (**H**), 80 μm (**I** and **J**), 300 μm (**K**). (**L**) Schematic depicting the spatial relationships of lymphatics (arrowheads) to nephron segments. All imaging from **E**–**K** representative of 5 regions of interest imaged across *n =* 2 kidneys.

To characterize lymphatic architecture in the healthy human kidney, we analyzed tissue samples from 4 deceased organ donors with minimal chronic damage (<10% interstitial fibrosis or tubular atrophy, Supplemental Table 1; supplemental material available online with this article; https://doi.org/10.1172/JCI168962DS1) (36). Intact tissue samples (<3 mm^3^) were immunolabeled using a D2-40 monoclonal antibody targeting podoplanin (PDPN) (37) and imaged using confocal or light-sheet fluorescence microscopy (LSFM) (16, 38). PDPN^+^ vessel networks were visualized in the human kidney cortex (Figure 1A and Supplemental Video 1), and antibody-omitted controls displayed minimal autofluorescence or nonspecific binding (Supplemental Figure 1A). Mapping vessel radius revealed a hierarchical network, with small lymphatics (radius ~3.5 μm) initiating in the cortex and converging into larger vessels (radius ~50 μm) at the corticomedullary junction (Figure 1B). The cells lining these vessels expressed prospero homeobox protein 1 (PROX1) (Figure 1C), a canonical LEC transcription factor (39, 40) but showed sparse expression of lymphatic vessel endothelial hyaluronan receptor 1 (LYVE1) (Figure 1D), a glycoprotein important for leukocyte entry into lymphatics (41).

To elucidate the microanatomical localization of lymphatics in the human kidney, autofluorescent tissue signals were captured alongside PDPN labeling. Large caliber lymphatics were observed adjacent to arteries at the corticomedullary junction branching into smaller cortical vessels (Supplemental Video 2). Colabeling with *Lotus tetragonolobus* lectin (LTL, proximal tubules) and uromodulin (UMOD, loop of Henle) revealed PDPN^+^ blind-ended lymphatics in the renal cortex (Figure 1E and Supplemental Video 3) and their absence from the medulla (Figure 1F). Despite previous reports of subcapsular lymphatics (42, 43), these were not detected in 3D reconstructions (Figure 1G) or optical z-sections (Supplemental Figure 1B), even with the kidney capsule intact. In the cortex, lymphatics followed UAE-I^+^ arterioles toward glomeruli (Figure 1H), extending terminal branches near megalin (LRP2^+^) proximal tubules (Figure 1I) and calbindin 1 (CALB1^+^) distal nephron epithelium (Figure 1J and Supplemental Video 4). Lymphatics converged toward the kidney hilum, adjacent to medullary *Dolichos biflorus* agglutinin (DBA^+^) collecting ducts (Figure 1K) and UMOD^+^ medullary tubules (Supplemental Figure 1C). A model summarizing these findings is presented in Figure 1L.

### Determination of the molecular identity of healthy human kidney lymphatics.

**Figure 2:**
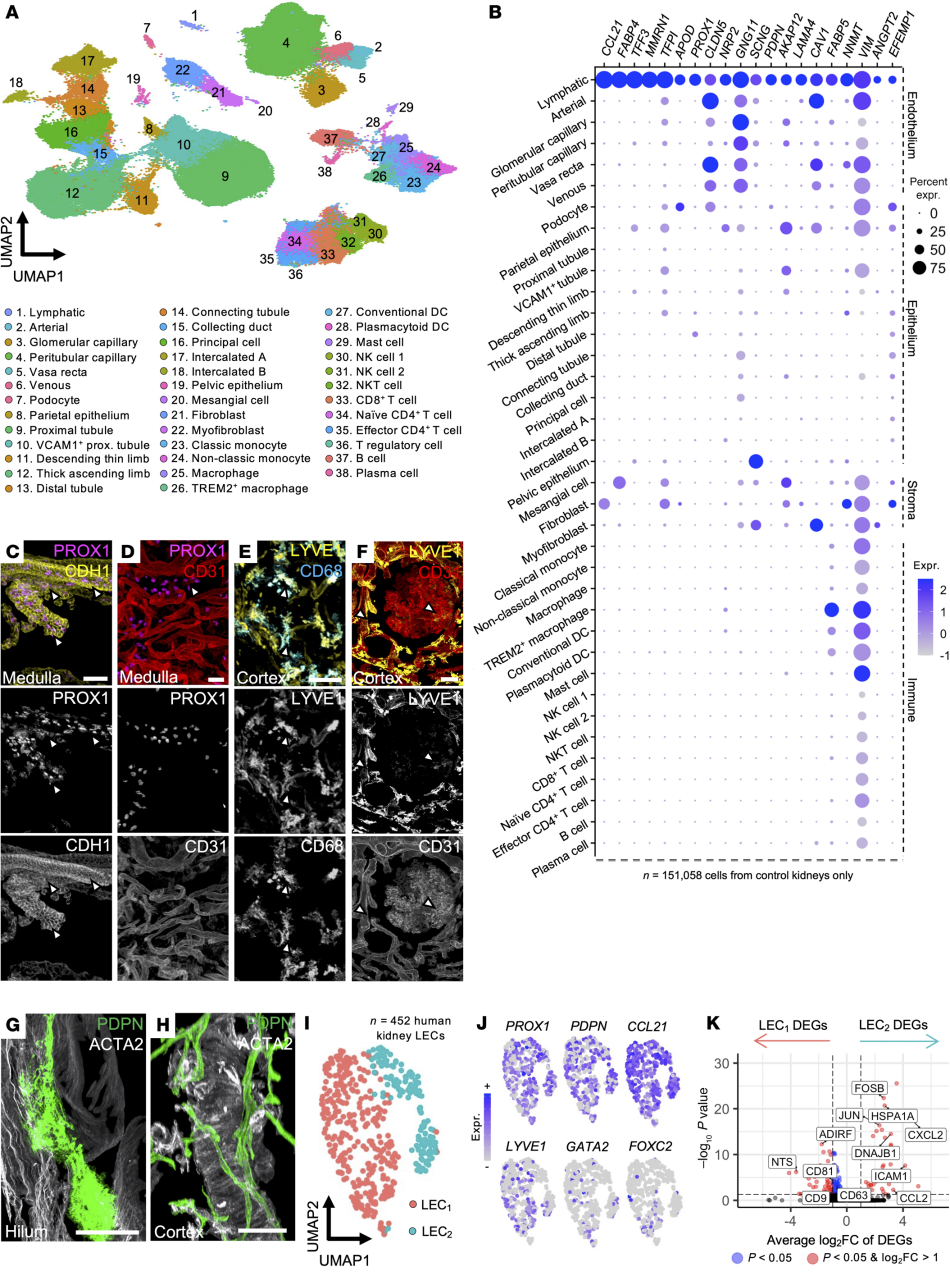
Profiling kidney lymphatics and their molecular signature through scRNA-Seq of human kidney tissue. (**A**) Uniform manifold approximation and projection (UMAP) of an integrated atlas of 217,411 cells, including 151,058 control cells from live biopsies or nephrectomies, 46,540 cells from different etiologies of graft injury, and 19,813 from chronic kidney disease. TREM, triggering receptor expressed on myeloid cells 2. (**B**) Dot plot of top 20 markers of lymphatic profiles across all control cell types in the atlas. Groupings for each cell type are shown on the right. (**C**–**F**) Analysis of nonlymphatic expression of PROX1 and LYVE1 using 3D imaging. Arrowheads show the expression of each marker relative to CDH1^+^ medullary tubules (**C**), CD31^+^ vasa recta (**D**), or CD68^+^ macrophages (**E**) and peritubular capillaries (**F**). Scale bars: 50 μm (**C**, **E**, and **F**), 30 μm (**D**). (**G** and **H**) Examination of ACTA2 expression relative to PDPN^+^ lymphatics (arrowheads) in the renal hilum (**G**) and cortex (**H**). Scale bars: 50 μm (**G**), 100 μm (**H**). (**I**) Subclustering analysis of *n =* 452 lymphatic endothelial cells (LECs) derived from human control kidney datasets reveals 2 transcriptionally distinct clusters, which we term LEC_1_ and LEC_2_. (**J**) Feature plots demonstrating expression of markers of all LECs (*PROX1*, *PDPN*), lymphatic capillaries (*CCL21*, *LYVE1*), and lymphatic collecting vessels (*GATA2*, *FOXC2*). (**K**) Volcano plot showing differentially expressed genes (DEGs) between the 2 lymphatic subclusters, with each point representing a gene. The *x* axis represents average log-fold change (log_2_FC), whereas the *y* axis represents –log_10_ of the adjusted *P* value of the Wilcoxon rank-sum test for differential expression. Blue dots represent genes that meet significance. Selected marker genes for each cluster are shown in boxes.

Because of the rarity of lymphatics in the human kidney relative to other cell types, isolating sufficient LECs for molecular profiling is challenging. To surmount this, we leveraged published scRNA-Seq data from 59 kidneys, supplemented with 5 new samples (Supplemental Figure 2A). This integrated dataset comprised 217,411 human kidney cells, with 151,038 control samples (living donor biopsies or unaffected regions of tumour nephrectomies) and 66,373 cells from diseased samples (chronic kidney disease, CKD) and kidney allograft injury; covering both chronic rejection and non-alloimmune etiologies) (Supplemental Figure 2B). All cell types were manually annotated (Supplemental Figure 2C), revealing 38 clusters (Figure 2A), including a transcriptionally distinct LEC cluster containing 700 cells.

From control samples, we curated a transcriptional signature of healthy kidney lymphatics (Supplemental Data 1), comprising 227 differentially expressed genes (DEGs) from 295 LECs. These genes were enriched for gene ontology (GO) terms associated with *lymphatic fate commitment* (GO:0060838, fold-enrichment > 100, FDR *=* 1.66 × 10^–2^) and *lymphangiogenesis* (GO:0001946, fold-enrichment = 67.4, FDR *=* 8.37 × 10^–3^). Canonical LEC markers were identified, including *PROX1* (log_2_FC = 2.97), *PDPN* (log_2_FC = 2.65), neuropilin 2 (*NRP2,* log_2_FC = 2.73), and C-C motif ligand *CCL21* (log_2_FC = 7.23) (Figure 2B). We also identified genes previously linked to kidney disease (44–46), such as fatty acid binding protein 4 *(FABP4*, log_2_FC = 5.69), trefoil factor 3 (*TFF3,* log_2_FC = 5.58), and angiopoietin 2 (*ANGPT2*, log_2_FC = 2.46) (Figure 2B).

Given the frequent use of PROX1 and LYVE1 to identify or target kidney lymphatics in preclinical studies (11, 14), we examined their expression within the human kidney in more detail. *PROX1* was detected not only in LECs, but also in loop of Henle and distal convoluted tubule clusters (Figure 2B), a finding validated by PROX1 and E-cadherin (CDH1^+^) immunolabeling of medullary tubules (Figure 2C) (47). In contrast to mouse data (38, 48), PROX1 was not detected in vasa recta at both the transcript (Figure 2B) and protein level (Figure 2D). LYVE1, meanwhile, was expressed by macrophages (Figure 2E) as reported in mouse (49) and human kidneys (50), and it was also detected in glomerular (Figure 2F) and peritubular capillary endothelium (Supplemental Figure 1, D–E).

To probe the phenotype of human kidney lymphatics, and whether the vessels we detected included smooth muscle–lined collecting vessels (51) as in the lungs (52) and skin and mesentery (53), we costained kidneys for PDPN and α-smooth muscle actin (ACTA2). Kidney lymphatics in both hilum and cortex lacked smooth muscle coverage (Figure 2, G and H). We corroborated this using subclustering analysis of our scRNA-Seq atlas, combining 295 LECs from healthy kidney with 157 additional cells from a recent study (54). This revealed 2 transcriptionally distinct LEC subclusters (Figure 2I), which expressed LEC capillary markers *PROX1,* *PDPN*, and *CCL21* (Figure 2J) with sparse *LYVE1* expression, consistent with our imaging data (Figure 1D). Only rare cells, not specific for either subcluster, expressed molecular markers of lymphatic valve endothelial cells, *GATA2* and *FOXC2* (Figure 2J). Differential expression analysis between the 2 capillary subclusters identified 129 DEGs (Supplemental Data 2). One subcluster was enriched for adipose signaling peptide neurotensin (*NTS,* log_2_FC = 3.60) (55), and the other expressed *CCL2* (log_2_FC = 3.30), *CXCL2* (log_2_FC = 4.22), and *ICAM1* (log_2_FC = 4.15) (Figure 2K), indicative of capillaries involved in immune cell egress.

### A multiorgan transcriptomic atlas reveals an organ-specific kidney lymphatic profile.

**Figure 3:**
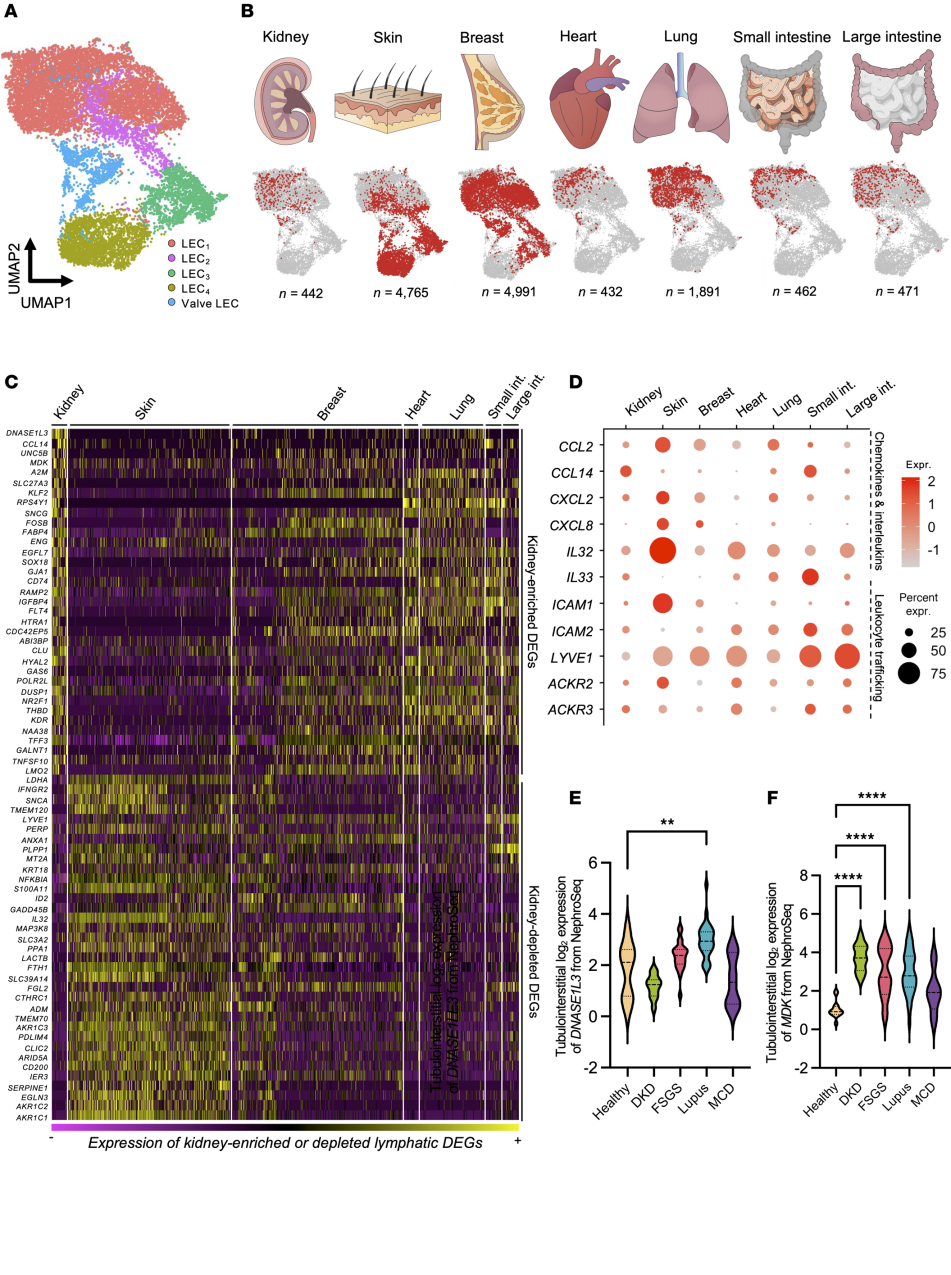
A single-cell atlas of human organ lymphatics reveals organ-specific molecular heterogeneity of kidney lymphatic endothelial cells. (**A**) Integrated UMAP featuring 13,454 cells from a total of 7 human organs incorporating kidney, skin, breast, heart, lung, small intestine, and large intestine. Unsupervised clustering resulting in 5 transcriptionally distinct clusters of lymphatic cells, which we designate LEC_1,_ LEC_2_, LEC_3_, and LEC_4_, all of which have capillary identity, and a fifth cluster representing valve LECs. (**B**) UMAPs highlighting the cells corresponding to each organ and where they are represented within the dataset. Based on this analysis, LEC_1_ and LEC_2_ are dominated by cells from visceral organs, including kidney, heart, lung, and intestines. Conversely, LEC_3_ and LEC_4_ are dominated by cells from superficial organs, the skin and breast tissue. All organs show cells mapping to valve LECs. (**C**) Heatmap showing the top 35 differentially expressed genes (DEGs) enriched in kidney lymphatic cells versus top 35 genes that have low expression by kidney lymphatics compared with other organs. (**D**) Dot plot of differentially expressed chemokines, interleukins, and immune trafficking receptors across lymphatics of different organs. (**E**) Expression of *DNASE1L3* and *MDK* (**F**) at the RNA level in the tubulointerstitium of patients within the publicly available NephroSeq database. Number of patients per condition are shown as follows for DNASE1L3: healthy (*n =* 8), diabetic kidney disease (DKD, *n =* 11), focal segmental glomerulosclerosis (FSGS, *n =* 22), lupus nephritis (*n =* 31, **P = 0.0013), minimal change disease (MCD, *n =* 9), and MDK: healthy (*n =* 14), DKD (*n =* 10, ****P < 0.0001), FSGS (*n =* 18, ****P < 0.0001), lupus nephritis (*n =* 31, ****P < 0.0001), MCD (*n =* 5). For both genes, significance values represent increase relative to healthy samples.

To investigate if, akin to their blood vascular counterparts, kidney lymphatics possess an organ-specific signature (56, 57), we created a multiorgan human LEC atlas by integrating our 452 kidney LECs with scRNA-Seq data of LECs from other organs, including skin (*n =* 4,765 cells) (58), breast (*n =* 4,991) (59), heart (*n =* 432) (60, 61), lung (*n =* 1,891) (62), and small and large intestine (*n =* 462 and 471, respectively) (63). The final dataset encompassed 13,454 LECs from 19 anatomical sites (Supplemental Figure 3A). We resolved 5 transcriptionally distinct subclusters (Figure 3A and Supplemental Data 3). Four subclusters expressed lymphatic capillary markers *CCL21* and *LYVE1*, while the fifth expressed lymphatic valve markers *FOXC2* and integrin alpha 9 (*ITGA9*) (Supplemental Figure 3B) (64). Visceral organ–derived LECs (kidney, heart, lung, intestines) were predominantly grouped within 1 subcluster (LEC_1_), whereas breast lymphatics were found in LEC_1_, LEC_2_, and LEC_3_, and skin lymphatics in LEC_2_, LEC_3_, and LEC_4_ (Figure 3B). This spatial segregation was reflected in predicted transcription factor activity (Supplemental Figure 3C).

Comparative analysis (Supplemental Data 4) identified 118 DEGs upregulated in kidney LECs compared with other organs (Figure 3C). The top kidney lymphatic-enriched genes included *DNASE1L3* (log_2_FC = 3.77, *P* = 3.24 × 10^–148^), the chemokine *CCL14* (log_2_FC = 3.03, *P* = 7.00 × 10^–59^), the netrin receptor *UNC5B* (log_2_FC = 2.26, *P* = 9.65 × 10^–29^), the growth factor midkine (*MDK,* log_2_FC = 1.98, *P* = 5.56 × 10^–21^), and the anti-protease α2 macroglobulin (*A2M,* log_2_FC = 1.80, *P* = 4.00 × 10^–33^). Most of these genes were also expressed by blood endothelia in the kidney, whereas *A2M* was also expressed by stromal cells (Supplemental Figure 3D). Among the 251 DEGs with lower expression in kidney LECs compared with those from other organs (Figure 3D) were *LYVE1* and the major neutrophil chemoattractant *CXCL8* (65), the latter of which was also absent from heart, lung, and intestinal LECs. Conversely, LECs in these visceral organs expressed the alarmin cytokine *IL33* (66), which was reduced in lymphatics of the skin and breast (Figure 3D and Supplemental Figure 3E).

To provide pathological context to the kidney lymphatic DEGs, we examined their expression in NephroSeq, a gene expression database of kidney diseases. *DNASE1L3* was significantly upregulated in the tubulointerstitium of patients with lupus nephritis (*n =* 31) compared with controls (*n =* 8, mean difference in log_2_ expressio*n =* 1.1 ± 0.34–1.9, *P* = 0.0013) (Figure 3E). Conversely, *MDK* was significantly upregulated in several inflammatory and metabolic kidney diseases, except for minimal change disease (Figure 3F).

Collectively, our analyses demonstrated that kidney LECs have an organ-specific molecular profile, enriched for *DNASE1L3*, *MDK*, and *CCL14,* with reduced expression of canonical immune trafficking markers such as *LYVE1* and *CXCL8*.

### Perturbation of kidney lymphatic architecture and endothelial junctional configuration in chronic transplant rejection.

Lymphangiogenesis has been observed during transplant rejection in both rodent models (23, 25, 31, 67) and humans (24, 26–28), but whether this is protective or promotes alloimmunity remains unclear. To investigate this in the human context, we profiled kidney transplants with chronic mixed rejection, a setting in which both donor-specific antibodies and T cells target HLA^+^ molecular expressed on tubular epithelial and blood endothelial cells.

**Figure 4:**
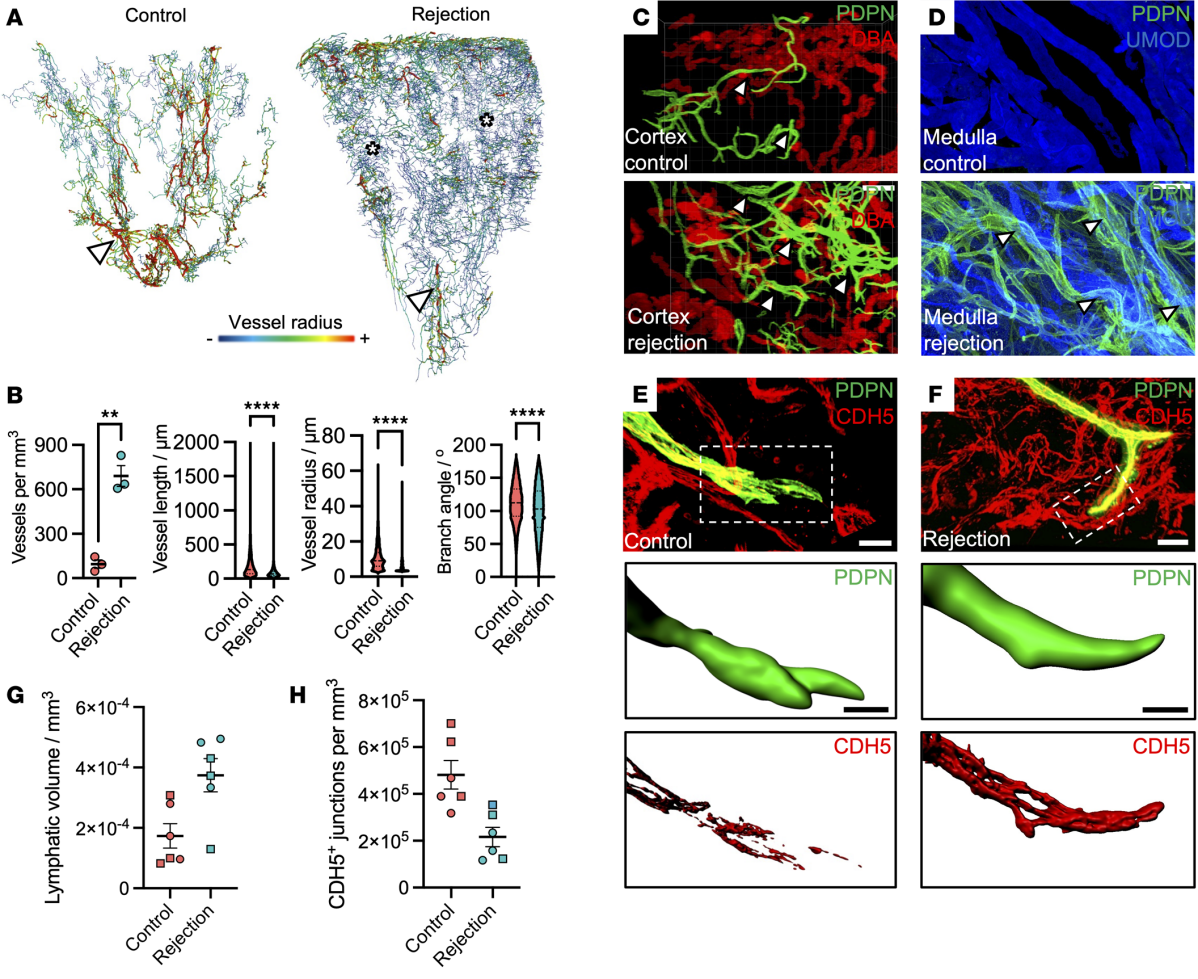
Structural remodeling of lymphatics in chronic transplant rejection. (**A**) 3D renderings of segmented lymphatic networks from donor kidneys and rejecting kidney allografts using LSFM; *n =* 3 samples per group. Vessel branch radii are color-coded: blue is smallest radius (<3.5 μm; asterisks) and red the largest (>18 μm; arrowheads). (**B**) Quantitative analysis of lymphatic branching architecture. Vessel metrics are shown per kidney (scatterplot, *n =* 3 per group) and pooled across vessels (violin plots, *n =* 75,036 vessels). Vessel density was significantly increased in rejection (95.12 ± 49.21 vs. 690.3 ± 121.6 vessels/mm^3^, **P = 0.0014, unpaired *t* test). Vessel length, radius, and branching angle distributions were significantly shifted in rejection (****P < 0.0001 for each; Kolmogorov–Smirnov tests). (**C** and **D**) Confocal imaging of PDPN^+^ lymphatic vessels (arrowheads) in cortex adjacent to DBA^+^ tubules (**C**) and medulla adjacent to UMOD^+^ tubules (**D**), showing lymphatic expansion in cortex and infiltration into medulla. Representative of 6 regions across *n =* 3 kidneys/group. Scale bars: 200 μm (**C**), 100 μm (**D**). (**E** and **F**) 3D reconstruction of CDH5^+^ lymphatic endothelial junctions in control (**E**) and rejecting (**F**) kidneys (*n =* 2 kidneys/group). Junctions identified within PDPN^+^ lymphatics using surface rendering in Imaris. Scale bars: 30 μm. Below: surface-rendered high-magnification views of lymphatic vessel blind ends from **E** and **F**, showing discontinuous CDH5^+^ “button-like” junctions in controls and continuous “zipper-like” junctions in rejection. Scale bars: 4 μm (control), 10 μm (rejection). (**G** and **H**) Quantification of total PDPN^+^ lymphatic vessel volume per field (**G**) and density of discontinuous CDH5^+^ junctions per mm³ of vessel volume (**H**). Each point represents a single image; circles, Repeat 1 and squares, Repeat 2. Rejecting kidneys showed increased lymphatic volume (mean difference = 2.01 × 10^–4^ ± 6.83 × 10^–5^ mm^3^) and reduced density of discontinuous junctions (mean difference = 265,674 ± 73,557 discontinuous junctions per mm^3^).

We analyzed 3 allografts with histological features consistent with chronic mixed rejection, including T cell– and antibody-mediated injury (Supplemental Table 2), and compared them with control kidneys obtained from nontransplanted donor organs. In rejecting allografts, the lymphatic vascular network exhibited marked disorganization, with loss of the hierarchical structure observed in controls (Figure 4A). Quantitative analysis revealed a 7-fold increase in mean lymphatic vessel density (95.12 ± 49.21 vs. 690.3 ± 121.6 vessels/mm^3^, *P* = 0.0014), accompanied by reductions in the distribution of vessel lengths (median difference = 132 vs. 68.4 μm, *P* = 0.0001), vessel radius (9.05 vs. 4.9 μm, *P <* 0.0001), and branching angle (112 versus 103, *P* < 0.0001) (Figure 4B and Supplemental Videos 5 and 6). Notably, lymphatic vessels also infiltrated the allograft medulla, a region devoid of lymphatics in healthy kidneys (Figure 4, C and D).

LEC-cell junctions are key regulators of immune cell trafficking. In homeostasis, these junctions form discontinuous “button-like” structures that facilitate leukocyte entry into lymphatics, whereas during chronic inflammation, they transition into continuous “zipper-like” formations that impair lymphatic drainage (68–70). Given the accumulation of infiltrating lymphocytes in chronically rejecting grafts (71–73), we hypothesized that altered lymphatic junctional architecture might be a feature of rejection. To assess this, we immunostained for vascular endothelial cadherin (CDH5), a key component of endothelial junctions (Supplemental Video 7), and used PDPN to distinguish lymphatics from blood vessels (Supplemental Figure 4). Discontinuous CDH5^+^ LEC junctions were quantified in both control (Figure 4E) and chronic rejection (Figure 4F) samples, and values were normalized to total lymphatic network volume (Figure 4G). We observed a reduction in disconnected (button-like) junctions in rejecting allografts compared with controls (Figure 4H, mean difference = 2.7 × 10^5^ ± 7.3 × 10^4^ CDH5^+^ junctions per mm^3^ lymphatic vessel), consistent with a shift toward a zipper-like configuration.

### Tertiary lymphoid structures form around lymphatics in chronic transplant rejection accompanied by altered intralymphatic and perilymphatic lymphocyte accumulation.

Given the structural perturbation of kidney lymphatics in rejecting allografts, we next examined their spatial relationship to organized immune responses within chronic rejection. A hallmark of alloimmunity is the formation of tertiary lymphoid structures (TLSs), ectopic lymph node–like aggregations of T cells and B cells, where follicular DCs and high endothelial venules (HEVs) also develop. TLSs facilitate local antigen presentation and lymphocyte activation, and they have been associated with progressive graft injury and dysfunction (74–79).

**Figure 5:**
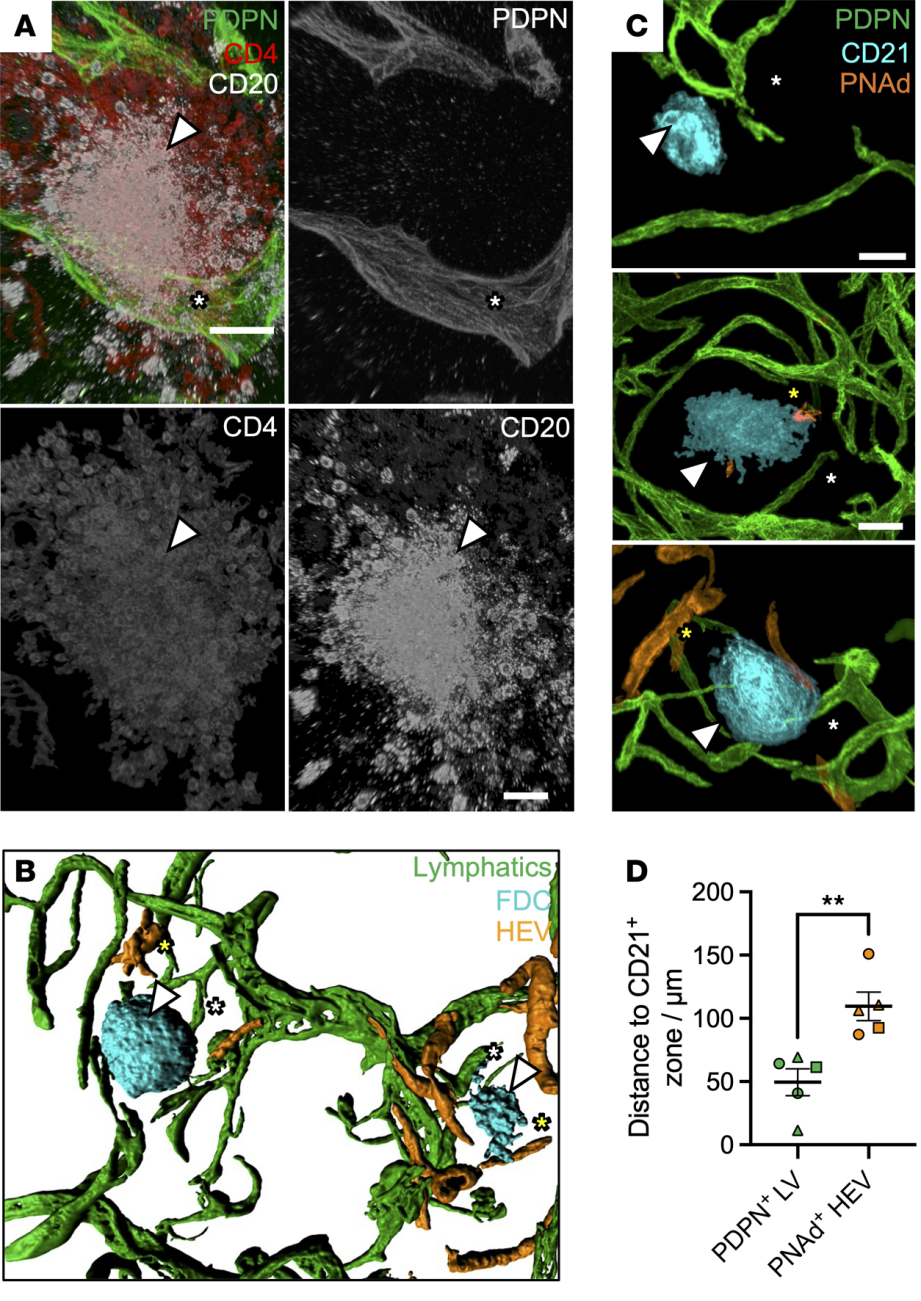
Spatial association between lymphatics and maturation of tertiary lymphoid structure. (**A**) Representative segmented confocal images of PDPN^+^ lymphatics (white arrowhead), CD20^+^ B cells, and CD4^+^ T cells in regions with evidence of ectopic lymphoid aggregation. A tertiary lymphoid structure (TLS) is shown (white asterisk). Representative image of 4 T cell– and B cell–rich TLSs taken from *n =* 2 rejecting allografts. Scale bar: 40 μm. (**B**) 3D rendering of TLS interconnected by lymphatics. Such interconnections (white arrowhead) were observed between TLSs in all (*n =* 3) rejecting allografts imaged. (**C**) Representative segmented confocal images of TLS, containing PDPN^+^ lymphatics (white arrow), CD21^+^ follicular DCs (FDCs) and peripheral lymph node addressin (PNAd^+^) high endothelial venules (HEVs). Nine TLSs were imaged across *n =* 3 rejecting allografts. Each image represents TLSs at different stages, with either HEVs absent (early stage; top image), scant (mid-stage; middle image), or present (late-stage, bottom image). Scale bar: 50 μm. (**D**) Comparison of distance between the CD21^+^ FDC core and lymphatic vessel (green) or HEVs (orange), with each data point representing an individual TLS imaged. Circles represent Repeat 1, squares Repeat 2, and triangles Repeat 3. Lymphatic vessels were significantly closer to CD21^+^ FDCs than HEVs (mean difference = 60.04, 95% CI = 24.33–95.76, **P = 0.0047, unpaired *t* test).

Using triple immunolabeling, we found PDPN^+^ lymphatics were observed close to CD4^+^ T cell– and CD20^+^ B cell–rich aggregates (Figure 5A) in 3 rejecting allografts, consistent with previous reports (26–28). To assess the relationship between lymphatics and TLS maturation, we examined PDPN^+^ lymphatics relative to CD21^+^ follicular DCs and peripheral lymph node addressin (PNAd^+^) HEVs, the latter serving as a marker of mature TLS (67, 80, 81). The lymphatic network in rejecting allografts interconnected multiple mature TLSs containing HEVs (Figure 5B and Supplemental Video 8). Such connections were not detected between CD31^+^ vessels (Supplemental Figure 5A). Spatiotemporal analysis revealed that all identified TLSs were near lymphatic vessels (Figure 5C) (*n =* 9/9, 100%), whereas only half contained HEVs (*n =* 5/9, 55.6%, *P =* 0.023). In mature TLSs with HEVs, PDPN^+^ LECs were significantly closer to the TLS core than HEVs (Figure 5D, mean distance = 49.53 ± 23.83 μm vs. 109.6 ± 25.13 μm, 95% CI = 24.33–95.76, *P =* 0.0047), suggesting that lymphatics are an early feature of TLS organization.

**Figure 6:**
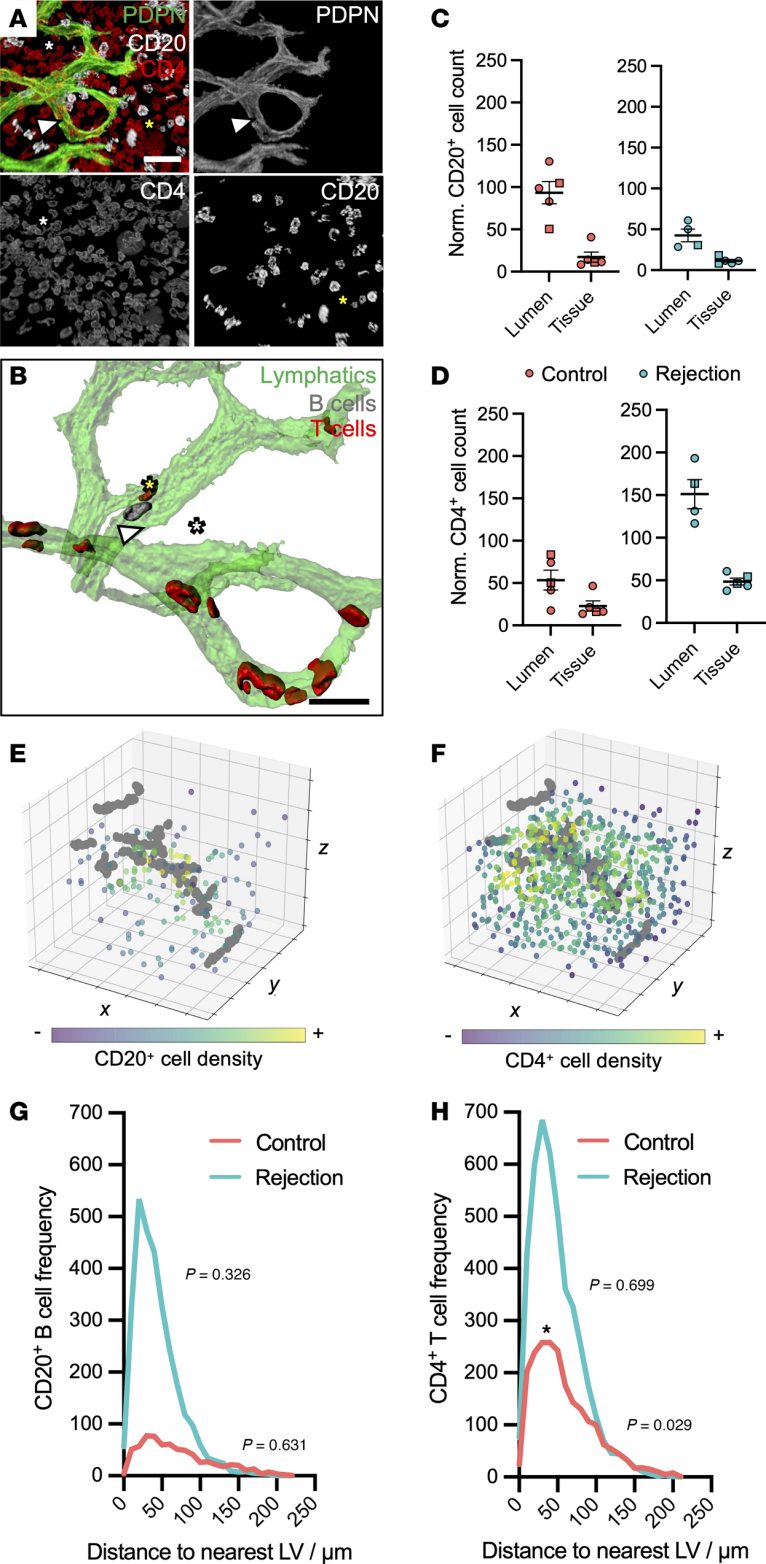
Molecular and spatial analyses indicate impaired T cell trafficking by kidney lymphatics in alloimmunity. (**A** and **B**) Segmented (**A**) and rendered (**B**) confocal images of PDPN^+^ lymphatics (white arrow), CD20^+^ B cells (yellow asterisk), and CD4^+^ T cells (white asterisk). In **B**, the transparency of rendered lymphatics is increased to visualize intraluminal B cells and T cells. Scale bars: 30 μm. (**C** and **D**) Number of intraluminal CD20^+^ B cells (**C**) or CD4^+^ T cells (**D**), normalized by volume, was quantified and compared with that of the tissue parenchyma. Each point represents 1 volume of interest imaged, with circles representing Repeat 1 and squares representing Repeat 2. Luminal CD20^+^ B cell density was higher than that of the tissue parenchyma in both control kidneys and rejecting allografts. A similar trend was observed for intraluminal CD4^+^ T cells, with a greater magnitude in increase in density within rejection. (**E** and **F**) Spatial point-pattern of perilymphatic CD20^+^ cell (**E**) or CD4^+^ cell (**F**) density, where lymphatic branch points represent gray dots and CD20^+^ cells are color-coded according to their density around the lymphatic network. (**G** and **H**) Histograms of CD20^+^ cell (**G**) or CD4^+^ T cell (**H**) frequency as a function of distance from the nearest lymphatic vessel. *P* values demonstrate whether lymphocytes are clustered around lymphatics greater than would be expected under complete spatial randomness. The only significant association observed was between CD4^+^ T cells and lymphatics in donor kidneys (*P = 0.029). All imaging data are representative of *n =* 5 imaging volumes, each acquired from *n =* 2 allografts with chronic mixed rejection and *n =* 2 donor controls.

To explore lymphatic-lymphocyte relationships beyond defined TLS regions, we performed 3D imaging and spatial quantification of PDPN^+^ lymphatics relative to CD20^+^ B cells and CD4^+^ T cells (Figure 6, A and B, and Supplemental Video 9). Intraluminal CD20^+^ B cell density was reduced by half in rejecting allografts compared with controls (Figure 6C), although total B cell numbers were equivocal, suggesting that this reflects increased lymphatic volume rather than changes in B cell abundance. In contrast, total intraluminal CD4^+^ T cells increased in rejecting kidneys, with a 3-fold increase in CD4^+^ T cell density (Figure 6D) relative to controls, a markedly higher density than was detected in the surrounding allograft parenchyma.

To further assess how lymphocyte position relative to lymphatics is altered in rejection, we performed spatial statistical analysis, by computing a normalized distance metric for each B cell (Figure 6E) and T cell (Figure 6F) to its nearest lymphatic vessel, and comparing this to a null model of random spatial distribution (82). CD20^+^ B cells showed no significant spatial association with lymphatics in either control kidneys (*n =* 703 cells; *P =* 0.631) or rejecting allografts (*n =* 2,963 cells; *P =* 0.326) (Figure 6G). However, CD4^+^ T cells (*n =* 2,149 cells across 2 controls) had a peak distribution within 0–100 μm from the nearest lymphatic vessel and were significantly enriched near lymphatic vessels compared with a random distribution (*P =* 0.029). This association was lost in rejecting allografts (*n =* 4,382 cells, *P =* 0.699) (Figure 6H), indicating disrupted T cell–lymphatic proximity in the context of chronic rejection.

### Molecular profiling reveals IFN-γ–driven coinhibitory remodeling and alloantibody targeting of allograft lymphatics.

Having established that lymphatics are structurally perturbed and spatially associated with immune aggregates in chronic rejection, we next investigated whether LECs in this setting exhibit an altered molecular profile. To do this, we first performed comparative transcriptomic analysis of LECs from healthy kidneys, rejection, and CKD (Supplemental Data 5–7).

**Figure 7:**
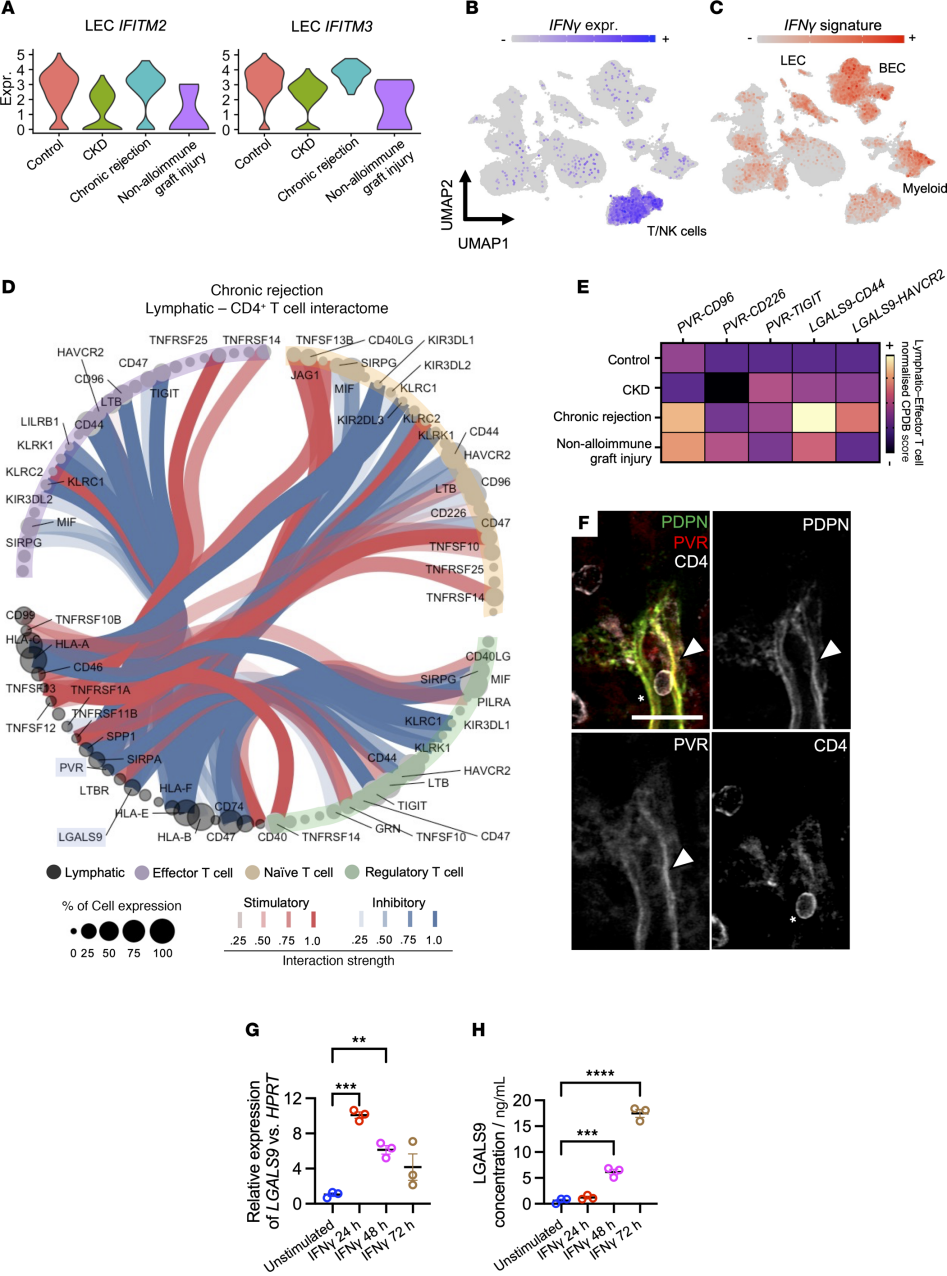
Interrogating kidney lymphatic–T cell crosstalk reveals a type 2 IFN-driven immunoinhibitory molecular landscape in alloimmunity. (**A**) Violin plots showing upregulation of IFN-inducible genes *IFITM2* and *IFITM3* in LECs from rejecting allografts. (**B**) UMAP of the scRNA-Seq data showing enrichment of IFN-γ within the T/NK cell cluster. (**C**) UMAP showing enrichment of an IFN-γ signature, including *IFNGR1*, *IFNGR2*, *IFITM2*, and *IFITM3*. (**D**) CellPhoneDB interaction map depicting predicted lymphatic-CD4^+^ T cell crosstalk in rejection. Inhibitory interactions (blue) include PVR and LGALS9; stimulatory interactions (red) are also shown. Node size reflects expression frequency; line intensity indicates interaction strength. Ligands of interest, *PVR* and *LGALS9*, are highlighted. (**E**) Heatmap of immune checkpoint interactions between LECs and effector CD4^+^ T cells across disease states. Color indicates normalized CellPhoneDB interaction score. All scores were normalized for each ligand-receptor pair. (**F**) Immunofluorescence validation of PVR expression on PDPN^+^ lymphatics (arrowhead) in rejecting kidneys (*n =* 2); CD4^+^ T cell shown in contact (asterisk). Scale bar: 30 μm. (**G**) IFN-γ stimulation of cultured human LECs increases LGALS9 levels at 24 and 48 hours (qPCR; ***P = 0.0002, **P = 0.0093, respectively) relative to HPRT. (**H**) LGALS9 protein secretion increased at 48 and 72 hours (ELISA; ***P = 0.0002, ****P < 0.0001, respectively) after IFN-γ stimulation of cultured human LECs. qPCR and ELISA experiments were repeated 3 times, and all assays were performed in duplicate, with each dot on the graph representing the mean data obtained for each repeat.

GO revealed that LECs from rejecting allografts were enriched for pathways related to the *negative regulation of viral process* (GO:0048525, fold-enrichment = 90.26, FDR *=* 5.95 × 10^–2^), including IFN-induced transmembrane proteins *IFITM2* (log_2_FC = 1.76, *P* = 5.89 × 10^–5^) and *IFITM3* (log_2_FC = 1.62, *P* = 6.86 × 10^–11^) (Figure 7A). IFN-γ was specifically enriched in T cells and NK cells in our scRNA-Seq dataset (Figure 7B), whereas other IFN types were not detected. We then examined an IFN-γ response signature — including levels of *IFITM2, IFITM3,* and the IFN-γ receptor subunits *IFNGR1* and *IFNGR2* — which was prominent in LECs and in blood endothelial cells and macrophages from rejecting allografts (Figure 7C). To contextualize this response, we compared the LEC profile in chronic rejection with that of HEVs, identified by enrichment for PNAd (*NTAN1*) and downregulation of Notch pathway genes *RBPJ* and *JAG1* (Supplemental Figure 5B) (83, 84). Unlike LECs, HEVs lacked lymphatic markers *PROX1* and *PDPN* (Supplemental Figure 5C). Instead, they expressed transcripts involved in leukocyte recruitment, activation, and regulation, such as *CXCL16*, fractalkine (*CX3CL1*), CD40, and IL-32 (Supplemental Figure 5D and Supplemental Data 8), highlighting a distinct immune regulatory profile compared with LECs.

We next explored potential ligand-receptor interactions between LECs and lymphocytes using CellPhoneDB (85). Predicted cell-cell communication was highest in rejecting kidneys compared with CKD or healthy controls (Supplemental Figure 6A), with most interactions occurring between LECs and T cell subsets (Supplemental Figure 6B). These included IFN-γ–IFNGR signaling from CD8^+^ T cells to LECs across both control and rejecting kidneys (Supplemental Figure 6C). Chemokine-based interactions included established axes such as *CCL21*, *CCL2*, and *ACKR2* (Supplemental Figure 7A), although CCL14/ACKR2 signaling with CD4^+^ effector T cells was reduced in rejection. Many chemokine receptors for ACKR2 ligands, including CCR2, CCR5, and CCR7, were expressed by T cells (Supplemental Figure 7B).

Notably, most of the remaining predicted interactions were coinhibitory in nature. These included LEC expression of poliovirus receptor (*PVR*) and galectin 9 (*LGALS9*), which suppress effector T cell responses via TIGIT and HAVCR2 signaling, respectively (86) (Figure 7D). While also present in CKD and non-alloimmune graft injury (Supplemental Figure 8, A–C), these interactions had higher signaling scores in chronic rejection (Figure 7E). Immunostaining confirmed PVR expression on PDPN^+^ lymphatics in direct contact with CD4^+^ T cells in rejecting allografts (Figure 7F). When stimulated by IFN-γ, blood endothelia express PVR and LGALS9 to dampen T cell responses (87, 88). To examine whether this was the case for LECs, we stimulated a human LEC line with recombinant IFN-γ. *LGALS9* transcripts were significantly upregulated after 24 hours (mean FC = 9.05, 95% CI = 5.37–12.73, adjusted *P =* 0.0002) and remained elevated at 48 hours (mean FC = 5.10, 95% CI = 1.42–8.78, adjusted *P =* 0.0093) (Figure 7G). Corresponding increases in LEC-secreted LGALS9 protein were observed at 48 hours (difference in mean concentratio*n =* 5.54 ng/mL, 95% CI = 3.26–7.83, adjusted *P =* 0.0002) and 72 hours (difference in mean concentratio*n =* 16.87 ng/mL, 95% CI = 14.58–19.16, adjusted *P <* 0.0001) (Figure 7H), confirming that LECs can acquire a coinhibitory profile in response to IFN-γ exposure.

**Figure 8:**
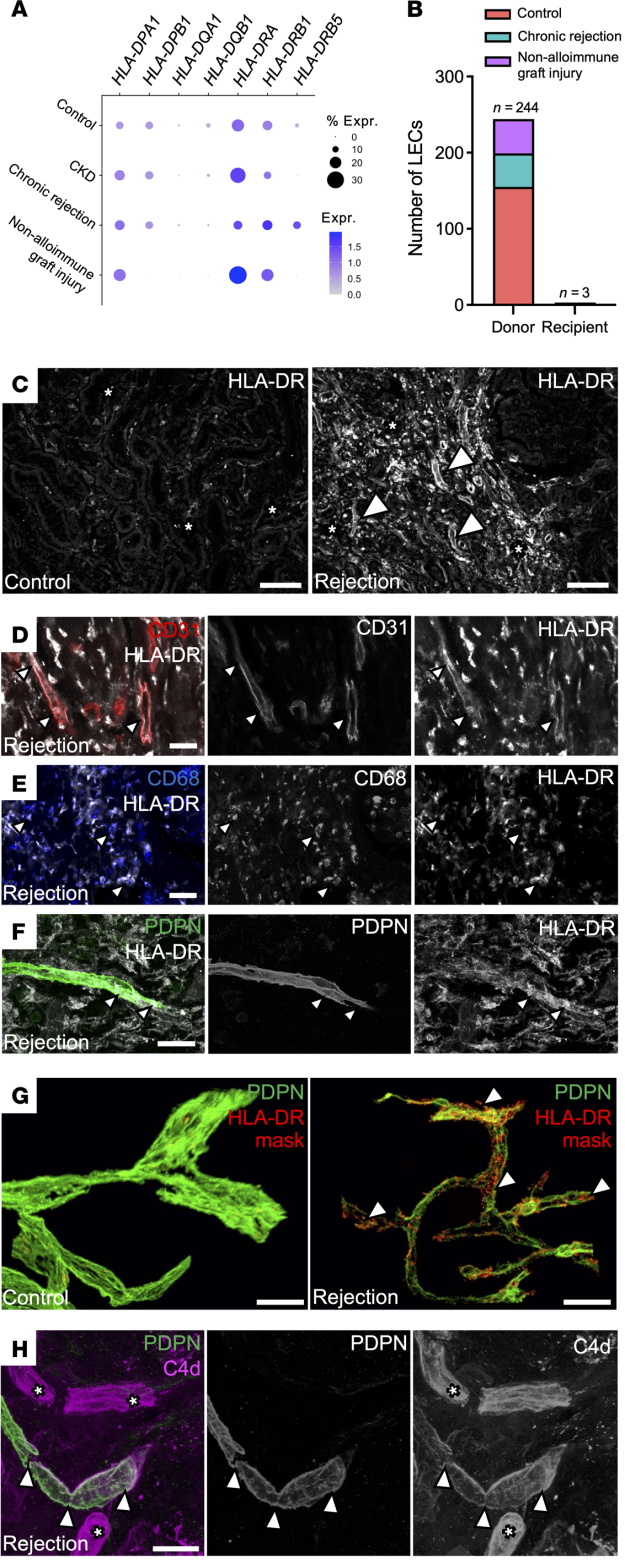
Donor lymphatics upregulate MHC class II molecules and represent a target for the alloimmune response. (**A**) Dot plot of the expression of transcripts encoding MHC class II molecules within lymphatics in the dataset. (**B**) Single nucleotide variant–based analysis of the origin of lymphatics in allograft tissues from the scRNA-Seq atlas. Cells are grouped by control, chronic rejection, or alternative causes of graft injury. (**C**) Representative optical z-sections from control and chronically rejecting renal tissue stained for HLA-DR. Isolated, discrete HLA-DR^+^ cells are shown with asterisks in both conditions, whereas in rejection there is also vascular staining (white arrowheads). Representative of 3 nonoverlapping fields of view per kidney, imaged across *n =* 2 kidneys per group. (**D**–**F**) 3D confocal images of HLA-DR expression (arrowheads) in CD31^+^ endothelia (**D**), CD68^+^ macrophages (**E**), and PDPN^+^ lymphatics (**F**). Images are representative of 5 regions imaged across *n =* 2 kidneys with chronic transplant rejection. All scale bars: 30 μm. (**G**) Representative 3D reconstructions of *n =* 2 transplant donor kidney tissues and *n =* 2 allograft tissues with chronic rejection stained using D2-40 and HLA-DR antibody. The HLA-DR signal is masked by D2-40 expression, such that only the signal inside lymphatics is visible. HLA-DR expression is observed in rejection (see white arrowheads). Three nonoverlapping fields of view per kidney were imaged. Scale bar: 50 μm. (**H**) 3D confocal images of C4d deposition, representative of 5 regions imaged across *n =* 2 kidneys with chronic transplant rejection. C4d deposition is observed in PDPN^+^ lymphatics (arrowheads) and presumptive blood capillaries (asterisks). Scale bar: 30 μm.

However, in solid organ transplantation, IFN-γ–induced expression of HLAs on endothelial cells can facilitate alloantigen presentation and antibody binding to donor vasculature (89, 90). Similarly, we found rejected allograft LECs also expressed *HLA-DP* and *HLA-DR* (Figure 8A). To determine whether lymphatics were of donor or recipient origin, we assessed genotype using single-nucleotide variant calling, and found a majority of LECs were donor derived, with a small recipient cell contribution (*n =* 3/247, 1.2%) (Figure 8B), consistent with a previous study of sex-mismatched renal allografts (91). Immunostaining for HLA-DR in chronic rejection (Figure 8C) demonstrated its expression on CD31^+^ blood endothelial cells (Figure 8D), CD68^+^ macrophages (Figure 8E) (92, 93), and PDPN^+^ lymphatics (Figure 8, F and G). Importantly, we detected complement factor C4d deposition, a histological hallmark of alloantibody-mediated complement activation, on PDPN^+^ lymphatic vessels in 2 rejecting allografts from patients with de novo donor-specific antibodies (Figure 8H). These HLA-DR^+^ lymphatic regions were surrounded by CD3^+^ T cells (Supplemental Video 10), suggesting coordinated alloantibody and T cell engagement. Together, these data demonstrate that LECs in chronic rejection acquire an IFN-γ–responsive, immune-inhibitory transcriptional phenotype, marked by coinhibitory ligand expression, HLA class II upregulation, and evidence of complement activation. 

## Discussion

Lymphatic vessels play a central role in maintaining fluid balance and immune homeostasis, yet their structural and molecular features in the human kidney remain underexplored. This gap is clinically relevant, as lymphangiogenesis occurs across a range of kidney diseases (11–14), and augmenting lymphatic function confers therapeutic benefit in preclinical models of kidney disease (94–96), hypertension (97–99), and acute kidney transplant rejection (29). Here, we combined 3D imaging of optically cleared tissue with scRNA-Seq to resolve the spatial architecture and molecular identity of lymphatics in the healthy human kidney and to interrogate their remodeling in chronic transplant rejection. Although previous studies have identified lymphatics in the kidney hilum and cortex (11–14), our 3D imaging approach yielded potentially new spatial insights, including a hierarchical arrangement of kidney lymphatics and the initiation of blind ends near proximal and distal tubular nephron segments, key sites of reabsorption and solute exchange between the urinary filtrate and blood. Using scRNA-Seq, we defined a transcriptional census of human kidney LECs, identifying expression of molecules previously characterized in other lymphatic beds but not in human kidney LECs, such as *FABP4* (100, 101) and *ANGPT2* (102–104).

A recent analysis has transcriptionally profiled a population of LECs in the lymph node (105). Our findings further extend the evidence for organ-specific heterogeneity of human lymphatics. Compared with lymphatics from barrier tissues such as skin, lung, and intestines, kidney LECs displayed reduced expression of genes encoding classical immune trafficking molecules like *CXCL8* and *LYVE1,* the latter confirmed at the protein level and also recently corroborated in mouse kidneys (106). Instead, kidney LECs express a repertoire of other molecules, including *DNASE1L3*, a molecule involved in extracellular DNA clearance and deficiency of which is implicated in lupus nephritis (107–109). Such findings could suggest tissue-specific adaptations of the lymphatic regulation of immunity and may inform future studies of immune-mediated kidney disease. Although lymphatic valve markers were sparsely detected, unlike in mouse kidneys (110), we identified transcriptional heterogeneity among kidney LECs, including a subpopulation enriched for *CCL2* and *CXCL2*. This is reminiscent of molecularly distinct and immune-interacting LEC subsets in the nasal mucosa (111, 112) and dermis (113). This heterogeneity may arise, in part, from microenvironment signals, such as IFN-γ, which drive context-dependent reprogramming of LECs in inflammation or cancer (114–116). We show that LECs upregulate PVR and LGALS9 in response to IFN-γ, echoing responses in the blood endothelium (87, 88) and supporting a paradigm in which the behavior of lymphatics is actively shaped by their surrounding milieu.

In kidney transplantation, lymphatics have been associated with improved graft survival, possibly through increased leukocyte clearance (27–29), but also with immune activation and fibrosis (23, 25, 31, 117, 118). Our findings challenge the notion that lymphangiogenesis is uniformly pathogenic. Although we observed lymphangiogenesis and proximity of these vessels to TLSs in rejecting allografts, we showed that allograft LECs acquire a tolerogenic transcriptional program driven by IFN-γ. LEC-derived immune-inhibitory ligands dampen effector T cell function in cancer (119, 120), neuroinflammation (121), and infection (69), and we confirmed the expression of 2 exemplar molecular candidates, PVR and LGALS9, at both the transcript and protein level.

However, this tolerogenic molecular program coincides with structural perturbations to allograft lymphatics. In rejection, lymphatics exhibited loss of hierarchical organization, infiltration into the medulla, and transformation of cell-cell junctions from button- to zipper-like morphology, changes known to impair fluid and cell transport (68–70). Building on previous studies in kidney (26, 27) and other inflammatory contexts (80, 122, 123), we identified TLSs of varying maturity positioned along lymphatic networks. Given the potential for in situ antigen presentation and T cell activation within the TLS (75, 77, 78, 124–127), and given the observed altered localization of CD4^+^ T cells within and around lymphatic vessels, it is tempting to speculate that lymphatic perturbation may contribute to CD4^+^ T cell retention within allografts, heralding the formation and maintenance of the TLS in chronic rejection. Additionally, we demonstrate that allograft LECs express HLA class II and show C4d deposition in patients with de novo donor-specific antibodies, consistent with alloantibody targeting and complement activation. Analogous injury to the blood vasculature (19) is well-characterized in transplant pathology (24), and donor lymphatics may thus represent a previously underappreciated target of alloimmune responses.

### Limitations.

This study has several limitations. First, our 3D imaging was cross-sectional and included a small number of fixed samples, restricting inference of dynamic events during transplant rejection. Second, and common to all scRNA-Seq studies of human tissues, our control tissues were derived from nontransplanted kidneys and tumour nephrectomies and are thus likely subject to inflammatory changes. We attempted to mitigate this by using samples with histological evidence of minimal chronic damage. Third, although we identified expression of coinhibitory ligands and evidence of alloantibody binding of kidney lymphatics, the downstream consequences on alloimmunity and graft function require further mechanistic study, which is challenging given the absence of an animal model that mimics the long-term sequalae of chronic mixed rejection, which occurred in our cohort of patients over decades to years, while enabling simultaneous genetic or pharmacological manipulation of LECs in a targeted manner.

### Conclusion.

Together, our data provide a comprehensive and multimodal view of the lymphatic vasculature in human kidney health and rejection. We propose that lymphatics acquire a tolerogenic, IFN-γ–driven phenotype during chronic rejection, but this is accompanied by structural disorganization and immune-associated perturbations. These findings point to a potentially new perspective on the role of lymphatic remodeling in transplantation, featuring a tolerogenic profile yet subject to alloimmune injury. This work lays the foundation for future studies exploring kidneys in health and disease and opens new avenues for therapeutic targeting of the lymphatic vasculature to improve the longevity of kidney transplants.

## Methods

### Sex as a biological variable.

Given the exploratory nature of 3D imaging and scRNA-Seq performed in this study and the limited kidneys available for 3D imaging analysis, sex was not considered as a biological variable.

### 3D imaging of human kidney lymphatics.

Human kidney tissue was fixed in 4% paraformaldehyde in PBS at 4°C overnight and stored in PBS with 0.02% sodium azide. A modified SHANEL protocol (128) was used for whole-mount immunolabeling, followed by optical clearing in benzyl alcohol/benzyl benzoate (1:2). Imaging was performed using an LSM880 upright confocal microscope (Zeiss) or custom-built mesoscale selective plane illumination microscope (mesoSPIM) (129). Image segmentation and 3D reconstruction were carried out in Imaris and Amira.

### Spatial analysis of lymphatic-lymphocyte relationships.

Binarized lymphatic networks were skeletonized in Fiji using BoneJ (130). CD4^+^ T cell and CD20^+^ B cell counts, centroids, and areas were obtained using 3D Objects Counter with no further preprocessing (131). The mean distance of each cell from the nearest point of the lymphatic network (*d*) was calculated using the cross-product 3D point-line distance:

 (Equation 1)
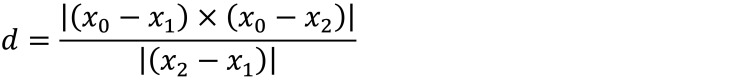


where *x*_1_ and *x*_2_ are the 2 closest adjacent nodes from the lymphatic 3D skeleton, found by minimizing cross–nearest neighbor distances, and *x*_0_ is the centroid of the cell of interest. To evaluate whether the cell distances were different from what would be expected by chance, within each region of interest, the CD4^+^ T cell and CD20^+^ B cell populations were randomly redistributed under complete spatial randomness for 20 simulations. A comparison was then made as to whether the measured mean cell-lymphatic distances fell within the 95% CIs obtained through the simulations under complete spatial randomness.

### scRNA-Seq and transcriptomic analysis.

Single-cell suspensions from fresh kidney explants were processed using the 10x Genomics Chromium 5′v2 kit and sequenced on an Illumina NovaSeq. Data were mapped to GRCh38 and processed using Scanpy and Seurat, using scVI (132) or Harmony (133) for integration. Cell identity was assigned via marker gene expression and assisted by CellTypist prediction. Differential expression was assessed using Wilcoxon rank-sum tests and GO term enrichment using PANTHER. To infer putative cell-cell interactions in scRNA-Seq data, the CellPhoneDB resource (85) was used. To generate the human lymphatic cell atlas, LECs were extracted from publicly available single-cell datasets across multiple organs and integrated using Harmony. SCENIC (134) was used to infer transcription factor activity across clusters. The NephroSeq database (v5, RRID:SCR_019050) was used to examine candidate genes by pulling data from its online browser.

### LEC stimulation assays.

Adult human dermal LECs (PromoCell, C-12217) were cultured in MV2 medium and treated with recombinant human IFN-γ (50 ng/mL) or unstimulated control medium for 24, 48, or 72 hours. *LGALS9* transcript levels were quantified by qRT-PCR and normalized to *HPRT* using the 2^–ΔΔCT^ method. Secreted LGALS9 protein in conditioned media was measured by ELISA (R&D Systems). Data are shown as fold-change relative to untreated controls. Assays were performed across 2 independent cell lines in triplicate.

### Statistics.

Statistical analyses were performed using GraphPad Prism unless otherwise specified. Data normality and variance were assessed using Shapiro-Wilk and Brown-Forsythe tests, respectively. For normally distributed data, comparisons between 2 groups used 2-tailed Student’s *t* test and 1-way ANOVA with Bonferroni’s post hoc tests for multiple groups. A *P* value less than 0.05 was considered significant. Data are presented as mean ± SD, with SEM shown for graphical error bars. Statistical methods for scRNA-Seq and spatial analyses are described separately.

### Study approval.

Use of human tissue was approved by NHS Blood & Transplant (NHSBT), the National Research Ethics Committee in the UK (21/WA/0388, NC.2018.010, NC.2018.007, REC 16/EE/0014), and the Royal Free London NHS Foundation Trust-UCL Biobank Ethical Review Committee (RFL B-ERC/B-ERC-RF, NC.2018.010; IRAS 208955). Written informed consent for research use of donated organs was obtained via NHSBT. Ethical approvals for public datasets are detailed in the original studies.

### Data availability.

Raw sequencing data for the 5 new human kidney scRNA-Seq samples have been made publicly accessible via the European Genome-phenome Archive (accession EGAD00001015631). Processed Seurat and h5ad files are available at Zenodo (https://doi.org/10.5281/zenodo.7566982). Code for data analysis is available at GitHub (https://github.com/daniyal-jafree1995/). Imaging data are available upon reasonable request. All raw data used to plot graphs, except for scRNA-Seq analyses, are provided within the Supporting Data Values file.

Full experimental details are provided in the Supplemental Methods, including reagents and protocols, in addition to the steps involved in computational analysis.

## Author contributions

DJJ, MRC, RM, and DAL conceived the study. Tissue acquisition and laboratory experiments were performed by DJJ, MKJ, KLP, CL, BM, LGR, LMR, WJM, BIL, LW, TW, SNM, JCC, and KXC. 3D image and scRNA-Seq data analyses were carried out by DJJ, BJS, BD, HM, AS, DM, NH, CJW, GP, CW, and SWS. Histopathological analysis and clinical data acquisition were performed by LH and RM. Guidance on IFN-γ stimulation assays was provided by CJW, LSKW, and TC. Project supervision and oversight were provided by PJDW, MAB, AG, PJS, MRC, RM, ASW, and DAL. DJJ wrote the first draft of the manuscript, which was refined by DAL, RM, and MRC. All authors contributed to editing and approved the final version for submission.

## Supplementary Material

Supplemental data

Supplemental data sets 1-2

Supplemental video 1

Supplemental video 2

Supplemental video 3

Supplemental video 4

Supplemental video 5

Supplemental video 6

Supplemental video 7

Supplemental video 8

Supplemental video 9

Supplemental video 10

Supporting data values

## Version 1 07/15/2025

In-Press Preview

## Version 2 09/16/2025

Electronic publication
