# Supplementary material for: Organ-specific features of human kidney lymphatics are disrupted in chronic transplant rejection
Source: J Clin Invest. 2025 Jul 15;135(18):e168962. doi: 10.1172/JCI168962 (PMC12435838; doi:10.1172/JCI168962)
Supplement: Supplemental data [file jci-135-168962-s283.pdf]

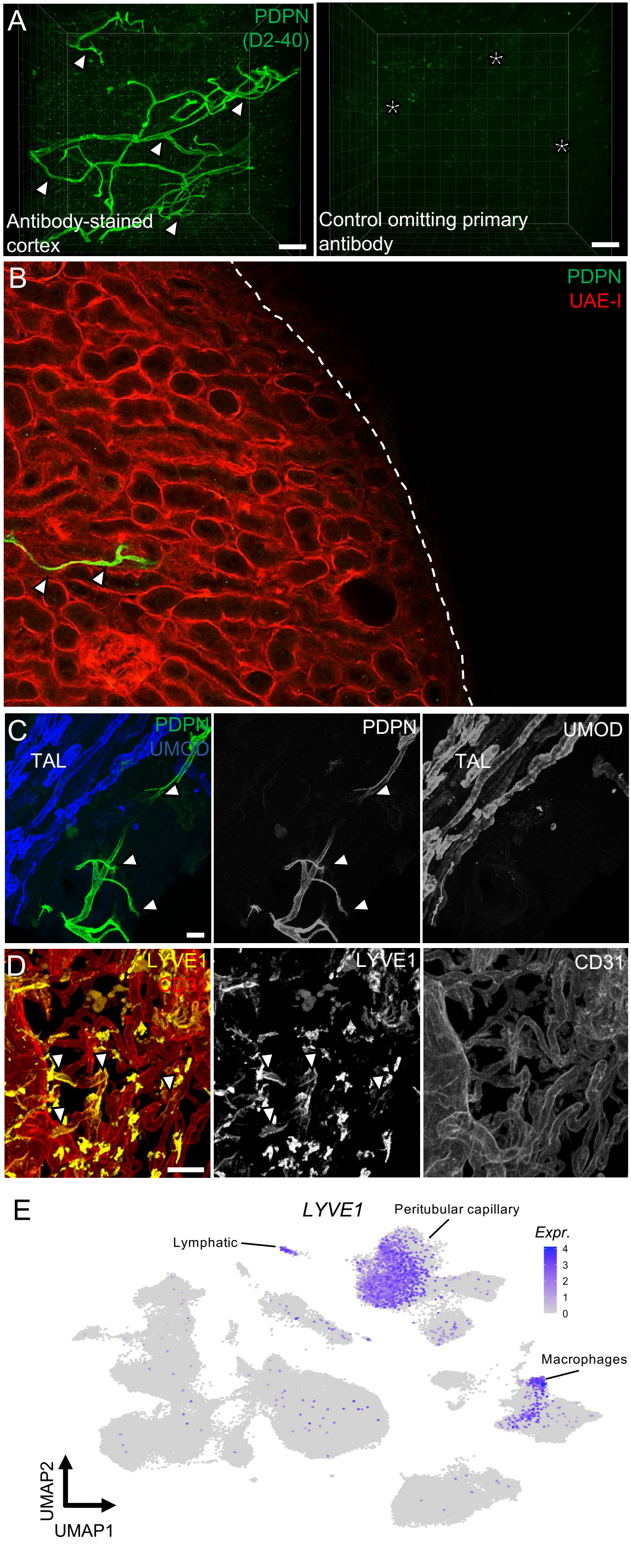
A
PDPN
(D2-40)
Antibody-stained cortex
Control omitting primary antibody
B
PDPN
UAE-I
C
PDPN
UMOD
TAL
PDPN
UMOD
TAL
D
LYVE1
CD31
LYVE1
CD31
E
LYVE1
Peritubular capillary
Lymphatic
Expr.
4
3
2
1
0
Macrophages
UMAP2
UMAP1

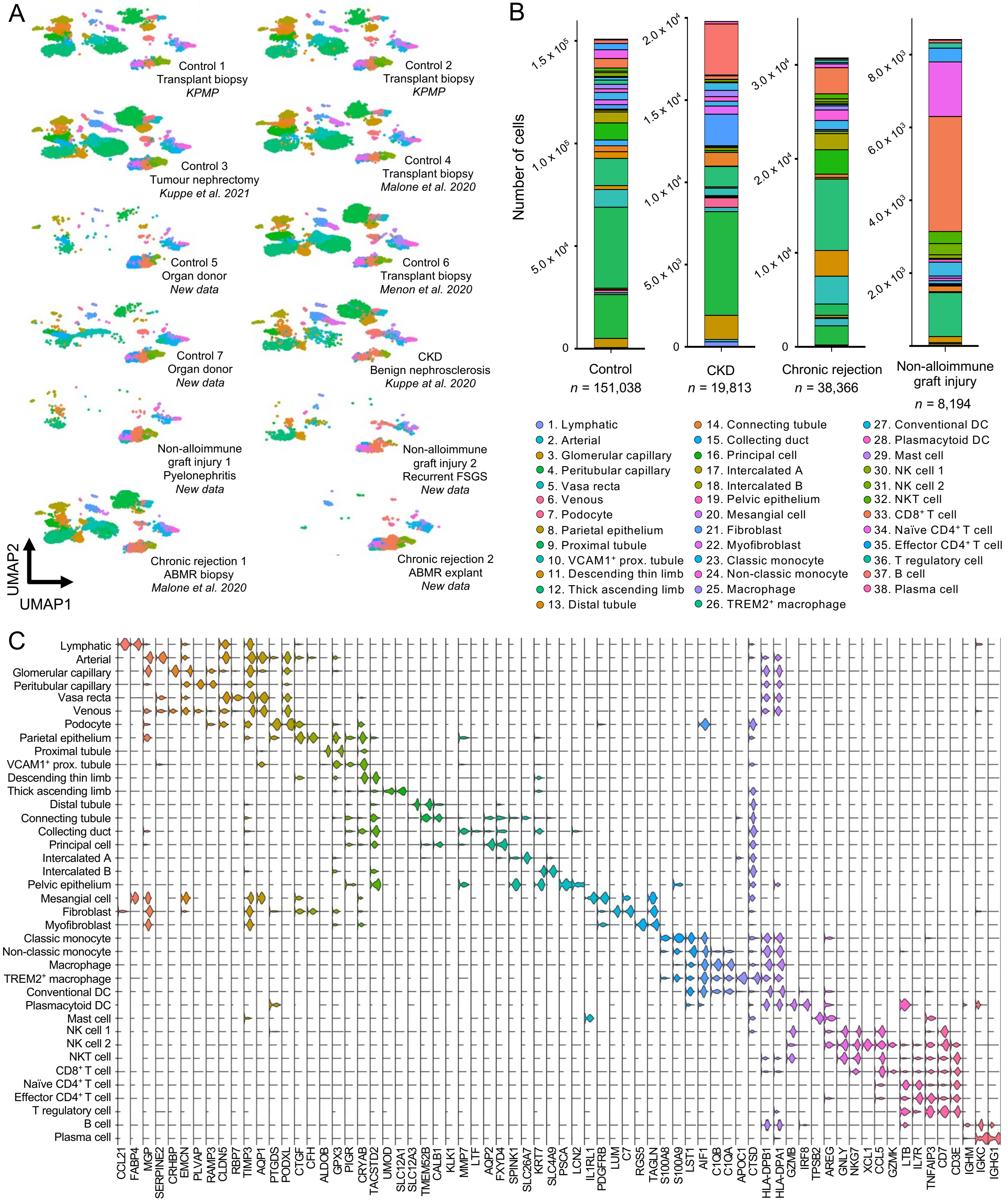
A
Control 1
Transplant biopsy
KPMP
Control 2
Transplant biopsy
KPMP
Control 3
Tumour nephrectomy
Kuppe et al. 2021
Control 4
Transplant biopsy
Malone et al. 2020
Control 5
Organ donor
New data
Control 6
Transplant biopsy
Menon et al. 2020
Control 7
Organ donor
New data
CKD
Benign nephrosclerosis
Kuppe at al. 2020
Non-alloimmune
graft injury 1
Pyelonephritis
New data
Non-alloimmune
graft injury 2
Recurrent FSGS
New data
Chronic rejection 1
ABMR biopsy
Malone et al. 2020
Chronic rejection 2
ABMR explant
New data
UMAP2
UMAP1
B
Number of cells
0
5.0 x 10^4
1.0 x 10^5
1.5 x 10^5
0
5.0 x 10^3
1.0 x 10^4
1.5 x 10^4
2.0 x 10^4
0
1.0 x 10^4
2.0 x 10^4
3.0 x 10^4
2.0 x 10^3
4.0 x 10^3
6.0 x 10^3
8.0 x 10^3
Control
n = 151,038
CKD
n = 19,813
Chronic rejection
n = 38,366
Non-alloimmune
graft injury
n = 8,194
1. Lymphatic
2. Arterial
3. Glomerular capillary
4. Peritubular capillary
5. Vasa recta
6. Venous
7. Podocyte
8. Parietal epithelium
9. Proximal tubule
10. VCAM1+ prox. tubule
11. Descending thin limb
12. Thick ascending limb
13. Distal tubule
14. Connecting tubule
15. Collecting duct
16. Principal cell
17. Intercalated A
18. Intercalated B
19. Pelvic epithelium
20. Mesangial cell
21. Fibroblast
22. Myofibroblast
23. Classic monocyte
24. Non-classic monocyte
25. Macrophage
26. TREM2+ macrophage
27. Conventional DC
28. Plasmacytoid DC
29. Mast cell
30. NK cell 1
31. NK cell 2
32. NKT cell
33. CD8+ T cell
34. Naïve CD4+ T cell
35. Effector CD4+ T cell
36. T regulatory cell
37. B cell
38. Plasma cell
C
Lymphatic
Arterial
Glomerular capillary
Peritubular capillary
Vasa recta
Venous
Podocyte
Parietal epithelium
Proximal tubule
VCAM1+ prox. tubule
Descending thin limb
Thick ascending limb
Distal tubule
Connecting tubule
Collecting duct
Principal cell
Intercalated A
Intercalated B
Pelvic epithelium
Mesangial cell
Fibroblast
Myofibroblast
Classic monocyte
Non-classic monocyte
Macrophage
TREM2+ macrophage
Conventional DC
Plasmacytoid DC
Mast cell
NK cell 1
NK cell 2
NKT cell
CD8+ T cell
Naïve CD4+ T cell
Effector CD4+ T cell
T regulatory cell
B cell
Plasma cell
CCL21
FABP4
MGP
SERPINE2
CRHBP
EMCN
PLVAP
RAMP3
CLDN5
RBP7
TIMP3
AQP1
PTGDS
PODXL
CTGF
CFH
ALDOB
GPX3
PIGR
CRYAB
TACSTD2
UMOD
SLC12A1
SLC12A3
TMEM52B
CALB1
KLK1
MMP7
LTF
AQP2
FXYD4
SPINK1
SLC26A7
KRT7
SLC4A9
PSCA
LCN2
IL1RL1
PDGFRB
LUM
C7
RGS5
TAGLN
S100A8
S100A9
LST1
AIF1
C1QB
C1QA
APOC1
CTSD
HLA-DPB1
HLA-DPA1
GZMB
IRF8
TPSB2
AREG
GNLY
NKG7
XCL1
CCL5
GZMK
LTB
IL7R
TNFAIP3
CD7
CD3E
IGHM
IGKC
IGHG1

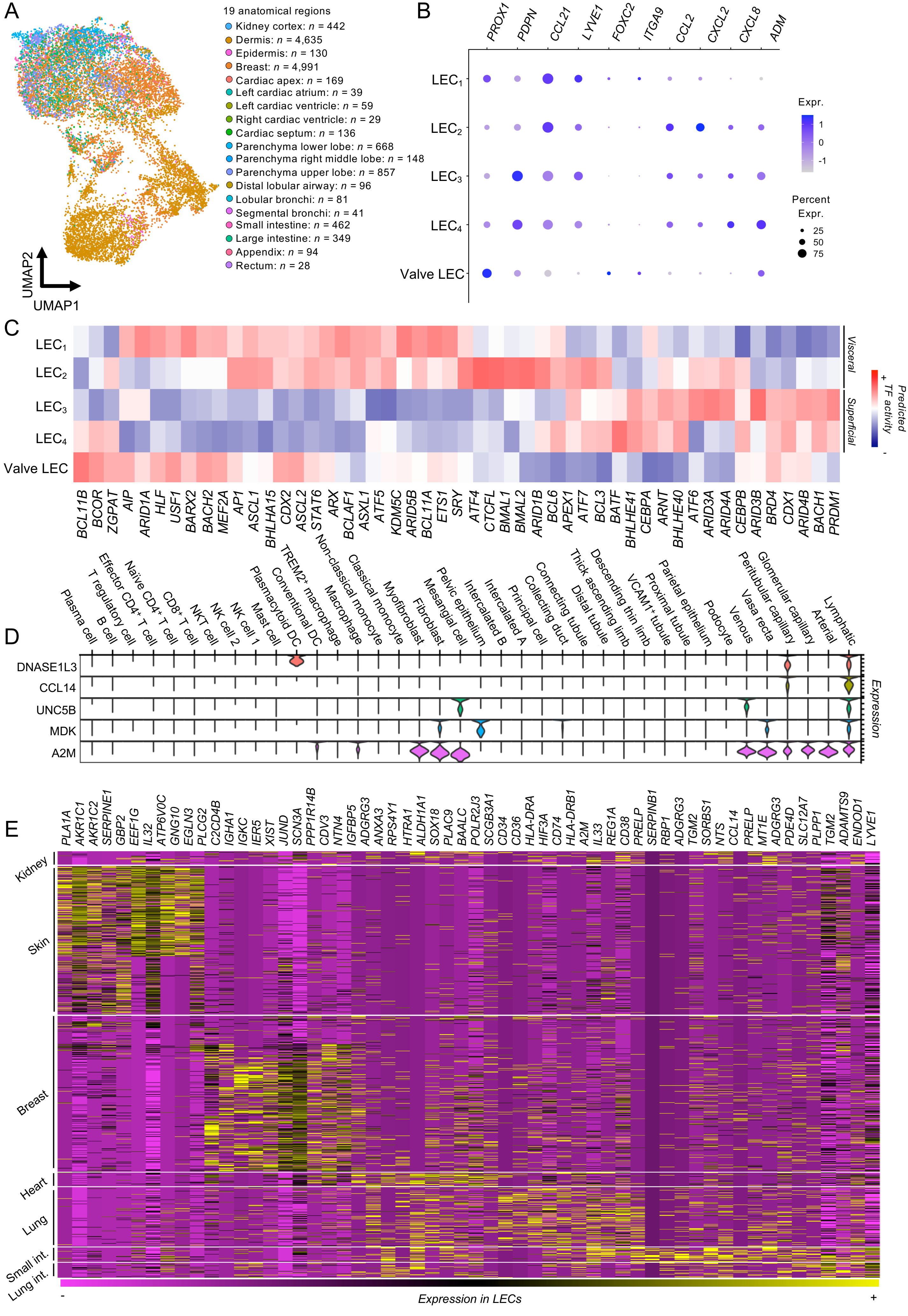
A
19 anatomical regions
Kidney cortex: n = 442
Dermis: n = 4,635
Epidermis: n = 130
Breast: n = 4,991
Cardiac apex: n = 169
Left cardiac atrium: n = 39
Left cardiac ventricle: n = 59
Right cardiac ventricle: n = 29
Cardiac septum: n = 136
Parenchyma lower lobe: n = 668
Parenchyma right middle lobe: n = 148
Parenchyma upper lobe: n = 857
Distal lobular airway: n = 96
Lobular bronchi: n = 81
Segmental bronchi: n = 41
Small intestine: n = 462
Large intestine: n = 349
Appendix: n = 94
Rectum: n = 28
UMAP2
UMAP1
B
PROX1 PDPN CCL21 LYVE1 FOXC2 ITGA9 CCL2 CXCL2 CXCL8 ADM
LEC1
LEC2
LEC3
LEC4
Valve LEC
Expr.
1
0
-1
Percent Expr.
25
50
75
C
LEC1
LEC2
LEC3
LEC4
Valve LEC
Visceral
Superficial
Predicted TF activity
+
-
BCL11B BCOR ZGPAT AIP ARID1A HLF USF1 BARX2 BACH2 MEF2A AP1 ASCL1 BHLHA15 CDX2 ASCL2 STAT6 ARX BCLAF1 ASXL1 ATF5 KDM5C ARID5B BCL11A ETS1 SRY ATF4 CTCFL BMAL1 BMAL2 ARID1B BCL6 APEX1 ATF7 BCL3 BATF BHLHE41 CEBPA ARNT BHLHE40 ATF6 ARID3A ARID4A CEBPB ARID3B BRD4 CDX1 ARID4B BACH1 PRDM1
D
Plasma cell
B cell
T regulatory cell
Effector CD4+ T cell
Naive CD4+ T cell
CD8+ T cell
NKT cell
NK cell 2
NK cell 1
Mast cell
Plasmacytoid DC
Conventional DC
TREM2+ macrophage
Macrophage
Non-classical monocyte
Classical monocyte
Myofibroblast
Fibroblast
Mesangial cell
Pelvic epithelium
Intercalated B
Intercalated A
Principal cell
Collecting cell
Connecting duct
Connecting tubule
Distal tubule
Thick ascending limb
Descending thin limb
VCAM1+ tubule
Proximal tubule
Parietal epithelium
Podocyte
Venous
Vasa recta
Peritubular capillary
Glomerular capillary
Arterial
Lymphatic
DNASE1L3
CCL14
UNC5B
MDK
A2M
Expression
E
PLA1A AKR1C1 AKR1C2 SERPINE1 GBP2 EEF1G IL32 ATP6V0C GNG10 EGLN3 PLCG2 C2CD4B IGHA1 IGKC IER5 XIST JUND SCN3A PPP1R14B CDV3 NTN4 IGFBP5 ADGRG3 ANXA3 RPS4Y1 HTRA1 ALDH1A1 SOX18 PLAC9 BAALC POLR2J3 SCGB3A1 CD34 CD36 HLA-DRA HIF3A CD74 HLA-DRB1 A2M IL33 REG1A CD38 PRELP SERPINB1 RBP1 ADGRG3 TGM2 SORBS1 NTS CCL14 PRELP MT1E ADGRG3 PDE4D SLC12A7 PLPP1 TGM2 ADAMTS9 ENDOD1 LYVE1
Kidney
Skin
Breast
Heart
Lung
Small int.
Lung int.
-
Expression in LECs
+

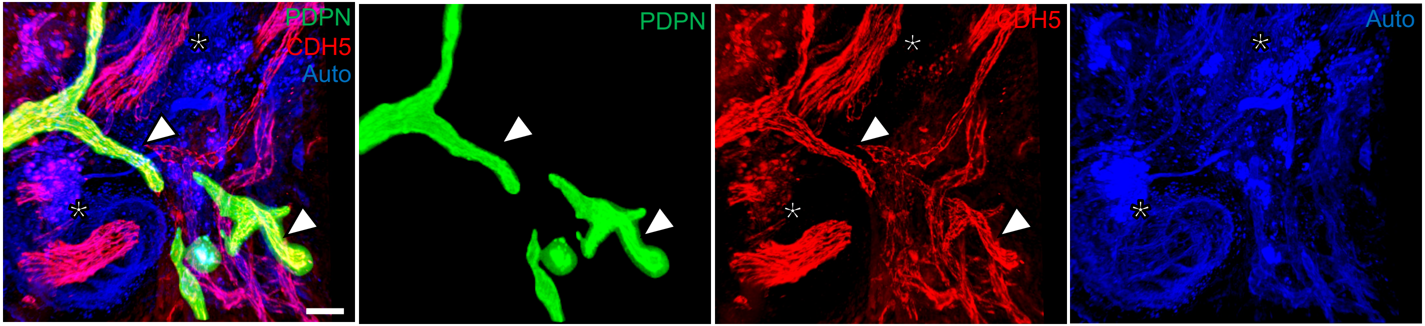
PDPN
CDH5
Auto
PDPN
CDH5
Auto

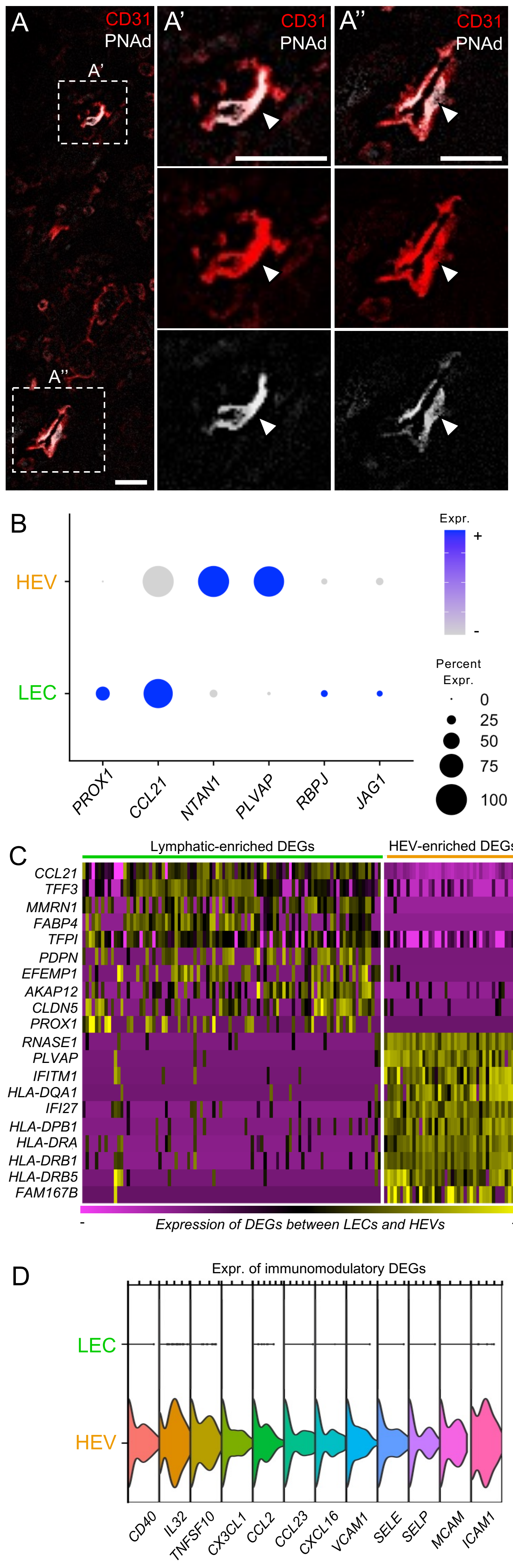

A
CD31
PNAd
A'
A''
B
HEV
LEC
PROX1
CCL21
NTAN1
PLVAP
RBPJ
JAG1
Expr.
+
-
Percent Expr.
0
25
50
75
100
C
Lymphatic-enriched DEGs
HEV-enriched DEGs
CCL21
TFF3
MMRN1
FABP4
TFPI
PDPN
EFEMP1
AKAP12
CLDN5
PROX1
RNASE1
PLVAP
IFITM1
HLA-DQA1
IFI27
HLA-DPB1
HLA-DRA
HLA-DRB1
HLA-DRB5
FAM167B
Expression of DEGs between LECs and HEVs
D
Expr. of immunomodulatory DEGs
LEC
HEV
CD40
IL32
TNFSF10
CX3CL1
CCL2
CCL23
CXCL16
VCAM1
SELE
SELP
MCAM
ICAM1

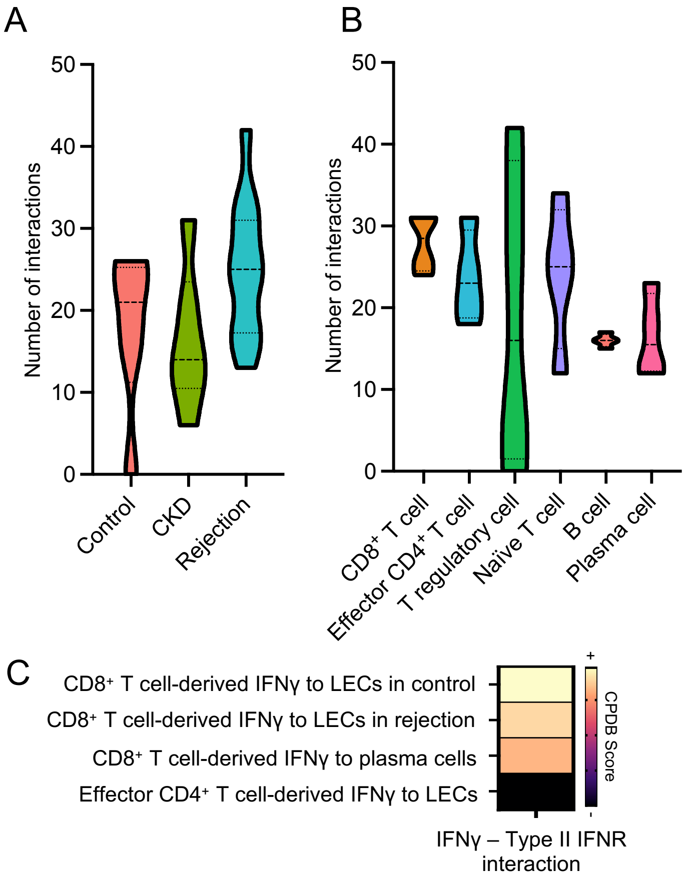
A
50
40
30
20
10
0
Number of interactions
Control
CKD
Rejection
B
50
40
30
20
10
0
Number of interactions
CD8⁺ T cell
Effector CD4⁺ T cell
T regulatory cell
Naïve T cell
B cell
Plasma cell
C
CD8⁺ T cell-derived IFNγ to LECs in control
CD8⁺ T cell-derived IFNγ to LECs in rejection
CD8⁺ T cell-derived IFNγ to plasma cells
Effector CD4⁺ T cell-derived IFNγ to LECs
+
-
CPDB Score
IFNγ – Type II IFNR interaction

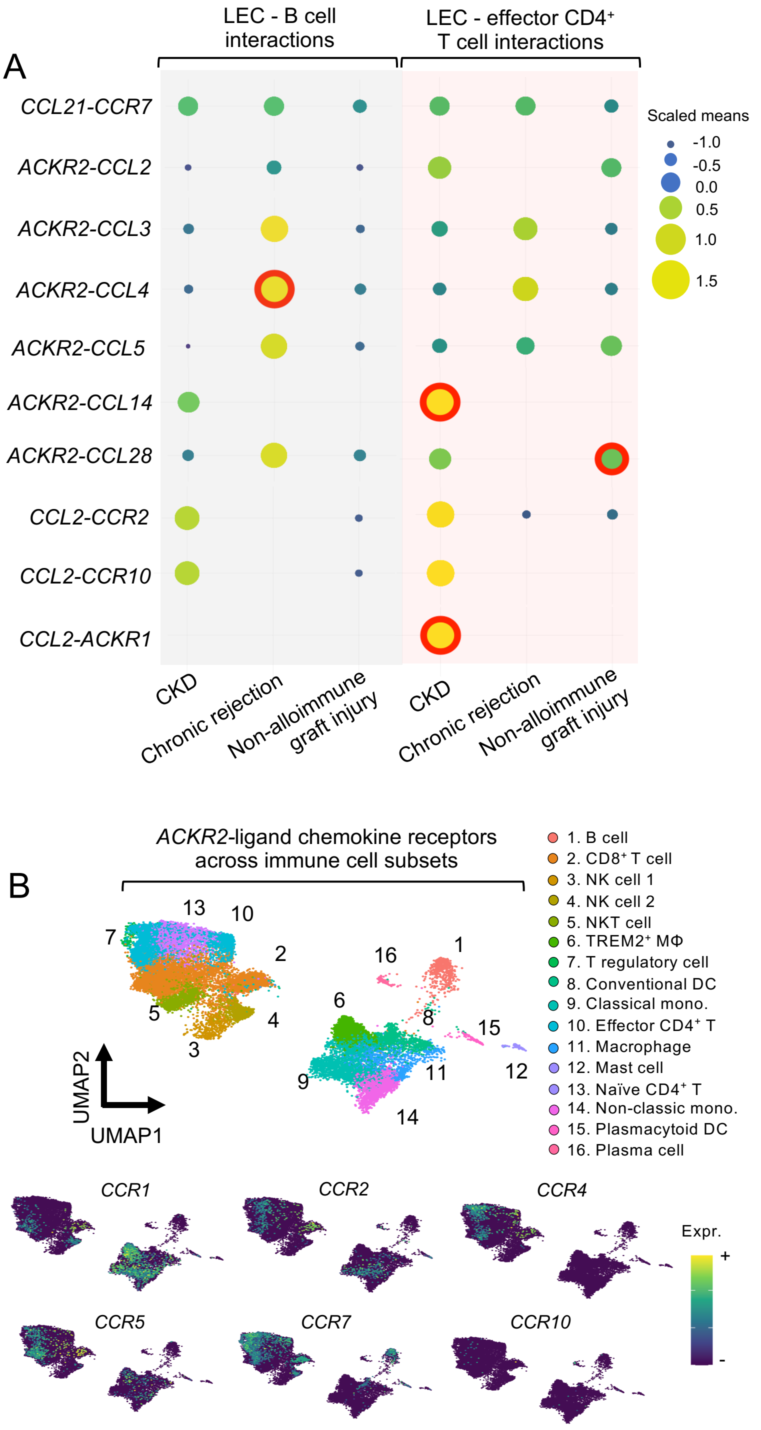

A
LEC - B cell interactions
LEC - effector CD4+ T cell interactions
CCL21-CCR7
ACKR2-CCL2
ACKR2-CCL3
ACKR2-CCL4
ACKR2-CCL5
ACKR2-CCL14
ACKR2-CCL28
CCL2-CCR2
CCL2-CCR10
CCL2-ACKR1
Scaled means
-1.0
-0.5
0.0
0.5
1.0
1.5
CKD
Chronic rejection
Non-alloimmune graft injury
CKD
Chronic rejection
Non-alloimmune graft injury
B
ACKR2-ligand chemokine receptors across immune cell subsets
UMAP2
UMAP1
1. B cell
2. CD8+ T cell
3. NK cell 1
4. NK cell 2
5. NKT cell
6. TREM2+ MΦ
7. T regulatory cell
8. Conventional DC
9. Classical mono.
10. Effector CD4+ T
11. Macrophage
12. Mast cell
13. Naïve CD4+ T
14. Non-classic mono.
15. Plasmacytoid DC
16. Plasma cell
CCR1
CCR2
CCR4
CCR5
CCR7
CCR10
Expr.
+
-

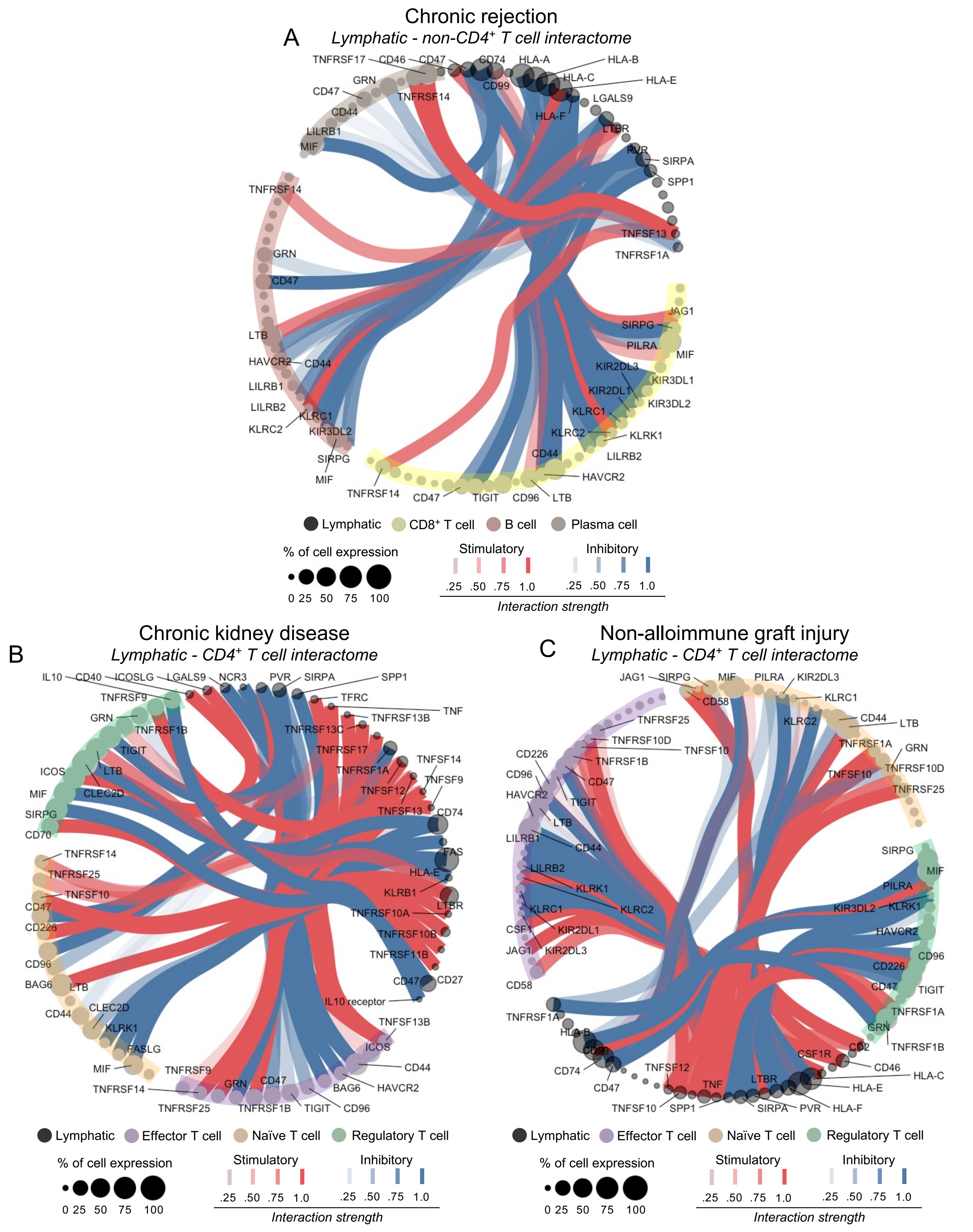

A
Chronic rejection
Lymphatic - non-CD4+ T cell interactome
TNFRSF17
CD46
CD47
CD74
HLA-A
HLA-B
HLA-C
HLA-E
GRN
CD47
TNFRSF14
CD99
CD44
HLA-F
LGALS9
LILRB1
MIF
LTBR
PVR
SIRPA
SPP1
TNFRSF14
TNFSF13
TNFRSF1A
GRN
CD47
JAG1
SIRPG
PILRA
MIF
LTB
HAVCR2
CD44
KIR2DL3
KIR3DL1
LILRB1
KIR2DL1
KIR3DL2
LILRB2
KLRC1
KLRC1
KLRC2
KLRC2
KLRK1
KIR3DL2
CD44
LILRB2
SIRPG
HAVCR2
MIF
TNFRSF14
CD47
TIGIT
CD96
LTB
Lymphatic
CD8+ T cell
B cell
Plasma cell
% of cell expression
0 25 50 75 100
Stimulatory
Inhibitory
.25 .50 .75 1.0
.25 .50 .75 1.0
Interaction strength
B
Chronic kidney disease
Lymphatic - CD4+ T cell interactome
IL10
CD40
ICOSLG
LGALS9
NCR3
PVR
SIRPA
SPP1
TNFRSF9
TFRC
TNF
GRN
TNFRSF1B
TNFRSF13C
TNFRSF13B
TIGIT
TNFRSF17
TNFSF14
ICOS
LTB
TNFRSF1A
TNFSF9
MIF
CLEC2D
TNFSF12
SIRPG
TNFSF13
CD74
CD70
TNFRSF14
FAS
TNFRSF25
HLA-E
TNFSF10
KLRB1
CD47
LTBR
CD226
TNFRSF10A
TNFRSF10B
CD96
TNFRSF11B
BAG6
LTB
CD47
CD27
CLEC2D
CD44
IL10 receptor
KLRK1
TNFSF13B
FASLG
ICOS
MIF
CD44
TNFRSF9
GRN
CD47
BAG6
HAVCR2
TNFRSF14
TNFRSF25
TNFRSF1B
TIGIT
CD96
Lymphatic
Effector T cell
Naïve T cell
Regulatory T cell
% of cell expression
0 25 50 75 100
Stimulatory
Inhibitory
.25 .50 .75 1.0
.25 .50 .75 1.0
Interaction strength
C
Non-alloimmune graft injury
Lymphatic - CD4+ T cell interactome
JAG1
SIRPG
MIF
PILRA
KIR2DL3
CD58
KLRC1
TNFRSF25
KLRC2
CD44
LTB
TNFRSF10D
TNFSF10
TNFRSF1A
GRN
CD226
TNFRSF1B
TNFRSF10D
CD96
CD47
TNFSF10
TNFRSF25
HAVCR2
TIGIT
LTB
LILRB1
CD44
SIRPG
MIF
LILRB2
PILRA
KLRK1
KIR3DL2
KLRK1
KLRC1
KLRC2
HAVCR2
CSF1
KIR2DL1
CD96
JAG1
KIR2DL3
CD226
CD58
CD47
TIGIT
TNFRSF1A
TNFRSF1A
GRN
HLA-B
TNFRSF1B
CD99
CD2
CSF1R
CD74
TNFSF12
CD46
HLA-C
CD47
TNF
LTBR
HLA-E
TNFSF10
SPP1
SIRPA
PVR
HLA-F
Lymphatic
Effector T cell
Naïve T cell
Regulatory T cell
% of cell expression
0 25 50 75 100
Stimulatory
Inhibitory
.25 .50 .75 1.0
.25 .50 .75 1.0
Interaction strength

Organ-specific features of human kidney lymphatics are disrupted in chronic transplant rejection

Daniyal J Jafree[1,2,3], Benjamin J Stewart[4,5], Karen L Price[1,2], Maria Kolatsi-Joannou[1,2], Camille Laroche[1,2], Barian Mohidin[1,2], Benjamin Davis[6], Hannah Mitchell[7], Lauren G Russell[1,2], Lucía Marinas del Rey[2,8,9], Chun Jing Wang[9], William J Mason[1,2], Byung Il Lee[10], Lauren Heptinstall[2,11], Ayshwarya Subramanian[12], Gideon Pomeranz[1,2], Dale Moulding[1], Laura Wilson[1,2], Tahmina Wickenden[1,2], Saif N Malik[1,2], Natalie Holroyd[13], Claire L Walsh[13], Jennifer C Chandler[1,2], Kevin X Cao[1,2], Paul JD Winyard[1,2], Adrian S Woolf[14], Marc Aurel Busche[10], Simon Walker-Samuel[13], Lucy SK Walker[9], Tessa Crompton[15], Peter J Scambler[1], Reza Motallebzadeh[2,8,9], Menna R Clatworthy[4,5]*, David A Long[1,2]*

[1]Developmental Biology & Cancer Research & Teaching Department, UCL Great Ormond Street Institute of Child Health, UCL, London, UK; [2]UCL Centre for Kidney & Bladder Health, UCL, London, UK; [3]UCL MB/PhD Programme, UCL, London, UK; [4]Molecular Immunity Unit, University of Cambridge, Cambridge, UK; [5]Wellcome Sanger Institute, Hinxton, Cambridge, UK; [6]Central Laser Facility, Science and Technologies Facilities Council, UK Research and Innovation, Didcot, Oxfordshire, UK; [7]Mathematical Sciences Research Centre, Queen's University Belfast, Belfast, UK; [8]Research Department of Surgical Biotechnology, Division of Surgery and Interventional Science, UCL, London, UK ; [9]UCL Institute of Immunity and Transplantation, UCL, London, UK; [10]UK Dementia Research Institute at UCL, London, UK; [11]UCL Department of Pathology, Royal Free Hospital, London, UK; [12]Department of Molecular Biology and Genetics, College of Arts and Science, Cornell University, Ithaca, NY, USA; [13]UCL Centre for Advanced Biomedical Imaging, London, UK; [14]School of Biological Sciences, Faculty of Biology Medicine and Health, University of Manchester, Manchester, United Kingdom; [15]Infection, Immunity and Inflammation Research and Teaching Department, UCL Great Ormond Street Institute of Child Health, UCL, London, UK;

***: Corresponding authors**

Professor David A Long

Professor in Paediatric Nephrology & Wellcome Trust Investigator in Science,

Developmental Biology and Cancer Research & Teaching Department,

UCL Great Ormond Street Institute of Child Health

30 Guilford Street, London, WC1N 1EH, UK

Tel.: +44(0)2079052615, Email: d.long@ucl.ac.uk

Professor Menna R Clatworthy

Molecular Immunity Unit,

University of Cambridge Department of Medicine,

MRC Laboratory of Molecular Biology,

Cambridge Biomedical Campus,

Francis Crick Avenue, Cambridge. CB2 0QH, UK

Tel.: +44(0)1223267279, Email: mrc38@cam.ac.uk

SUPPLEMENTARY FIGURE LEGENDS

Fig.S1. Further characterization of kidney lymphatic molecular markers and cellular relationships

(A) Representative 3D reconstruction of three human cortical tissues stained for a PDPN monoclonal antibody (clone: D2-40) showing lymphatic vessels (white arrowheads) as compared to controls in which primary antibodies were omitted, resulting in no labelling of vessel structures. Some areas exhibited non-specific fluorescence / background immunoreactivity. Imaging is representative of three non-overlapping fields of view captured per donor kidney. Scale bars: 150 µm. (B) Representative *z*-section from confocal image stack as shown in Figure 1G, showing the extent to which PDPN$^+$ lymphatic vessels, shown with white arrowheads, extend towards the peripheries of the kidney cortex (shown with a white dashed line). (C) Representative 3D reconstruction of juxtamedullary regions from two human transplant donors labelled for PDPN and the thick ascending limb epithelial marker, uromodulin (UMOD). 3D reconstructions revealed that lymphatic vessels (LV) run adjacent to, but do not intermingle with medullary bundles of TAL tubules. Scale bar: 100 µm. (D) Analysis of non-lymphatic expression LYVE1 using 3D imaging within CD31$^+$ peritubular capillaries, shown with white arrowheads. Scale bar = 50 µm. (E) UMAP of the scRNA-seq dataset from control kidneys showing the expression of *LYVE1*. The UMAP corresponds to that in Figure 2A, and shows *LYVE1* expression in lymphatics, peritubular capillary endothelium and myeloid cells within the human kidney.

Fig.S2. Generating a human kidney single-cell transcriptomic atlas including alloimmune and non-alloimmune pathologies

(A) UMAP of the kidney cell atlas coloured by cell type and partitioned by dataset. Samples included non-rejection transplant biopsies (Control 1: 7,259 cells, Control 2: 9,785 cells, Control 4: 38,850 cells and Control 6: 22,592 cells), non-tumorous regions of tumour nephrectomies (Control 3: 58,934 cells, chronic kidney disease due to benign nephrosclerosis

(CKD): 58,934 cells), declined organ donor tissues (Control 5: 3,877 cells , Control 7: 9,741 cells) and surgically explanted allografts (Non-alloimmune graft injury 1: 6,063 cells, Non-alloimmune graft injury 2: 2,131 cells, Chronic rejection 1: 34,067 cells, Chronic rejection 2: 4,299 cells). (B) Stacked bar charts representing the relative proportions of each annotated cell type across the four study groups). (C) Stacked violin plot showing top 2 differentially expressed marker genes (*y* axis) by cell type (*x* axis), calculated using Seurat FindAllMarkers.

Fig.S3. Anatomical and organ-specific molecular heterogeneity within the human organ lymphatic atlas

(A) Integrated UMAP, as shown in Figure 3A, featuring 13,454 cells from a total of seven human organs, including kidney, skin, breast, heart lung, small intestine and large intestine. The cells here are grouped by the 19 anatomical regions from which the cells were acquired. (B) Dot plot showing marker genes of each LEC subtype. Expression of *PROX1* and *PDPN* confirms the lymphatic identity of all cells within the dataset. *CCL21* and *LYVE1* demarcate capillary lymphatic cells and are enriched in all clusters except for valve LECs, which instead are enriched for *FOXC2* and *ITGA9*. The remaining clusters have variable expression of the chemokines *CCL2, CXCL2*, *CXCL8,* and the peptide hormone *ADM.* (C) Heatmap showing predicted cell cluster-enriched transcription factor (TF) activity based on cluster-enriched DEGs, assessed using SCENIC analysis. Red indicates higher relative predicted activity whereas blue indicates lower relative predicted activity. TFs are cluster depending on whether the organ sampled was viscerally or superficially located. (D) Violin plot of cell type-specific expression of the 5 top differentially expressed genes by kidney lymphatics, across other cell types within the kidney scRNA-seq atlas (E) Heatmap showing top 10 marker genes of each non-kidney organ within the scRNA-seq dataset of human organ lymphatics.

Fig.S4. Identification of endothelial cell-cell junctions in three-dimensional imaging data of human kidney

3D reconstruction of confocal imaging of kidney cortical lymphatics (white arrowheads) in chronic transplant rejection tissues, representative of three images each acquired from *n* = 2 rejecting allografts. Endothelial cell junctions were labelled with CDH5. Arterioles in juxtaposition with lymphatics are shown with white asterisks. The 488nm channel was used to image autofluorescence and capture tissue architecture. Scale bar: 30 µm.

Fig.S5. Identification and characterization of high endothelial venules 3D imaging and scRNA-seq data of kidney transplant rejection

(A) Representative *z*-section of three confocal microscopy *z*-stacks acquired from *n* = 2 kidneys with chronic transplant rejection, stained for CD31 and the high endothelial venule (HEV) marker PNAd. Within the image, two HEV structures are shown, individually magnified on the panels on the right, with each panel labelled accordingly. $PNAd^{+}$ $CD31^{+}$ endothelium is shown with white arrowheads. Scale bars: 50 µm. (B) Dot plots demonstrating molecular characterisation of HEV endothelium with kidney transplant rejection scRNA-seq data. Lymphatic endothelial cells (LEC) are used as a comparison, and are enriched for *PROX1* and *CCL21*, whereas *NTAN1* and *PLVAP* are enriched in HEV cells. Compared to lymphatics, HEV endothelium has low expression of the Notch pathway molecules including *RBPJ* and *JAG1*. (C) Heatmap showing differentially expressed genes (DEG) between LECs and HEVs. Of note, a variety of MHC Class II encoding transcripts are upregulated in HEV endothelium as compared to lymphatics. (D) Stacked violin plot showing immunologically relevant differentially expressed genes between lymphatic endothelium and $CCL21^{+}$ $PNAd^{+}$ blood endothelium across rejecting allograft scRNA-seq data. Immune activating candidates (*CD40, IL32, TNFSF10, CX3CL1*), chemokines (*CX3CL1, CCL2, CCL23, CXCL16*) and immune cell adhesion molecules (*VCAM1, SELE, SELP, MCAM, VCAM1*) are all enriched in HEV cells.

Fig.S6. Cell-cell interaction analysis of lymphatics in human kidney single-cell RNA sequencing data

(A) Violin plot showing the total number of CellPhoneDB-computed cell-cell interactions between lymphatics and adaptive immune cell subsets within single-cell RNA sequencing (scRNA-seq) data of control, chronic kidney disease (CKD), and rejection tissues. (B) shows the interactions as A but are partitioned by the subtype of lymphocyte. (C) Statistically significant CellPhoneDB scores for lymphocyte-derived IFNγ interacting with lymphatics or other cell types within the dataset.

Fig.S7. Analysis of chemokine signalling mediated by kidney lymphatics in health and disease

(A) CellPhoneDB dot plot of scRNA-seq data demonstrating top 10 chemokine interactions between lymphatics and B cells or effector $CD4^+$ T cells, partitioned by disease aetiology. Dot size represents the scaled mean expression of the interaction, and those encircled with a red ring are deemed statistically significant by CellPhoneDB. (B) UMAP of all immune cell subsets with feature plots showing expression of ACKR2 ligands across these cell types.

Fig.S8. Visualization of immune-stimulatory and immune-inhibitory interactions mediated by kidney lymphatics in health and disease

(A-C) Circle plot of the transcriptional ‘interactome’, or putative cell-cell communication, between lymphatics and distinct immune cell subsets identified in the scRNA-seq dataset, computed between lymphatics and non-$CD4^+$ T cells in chronic rejection (A), lymphatics and $CD4^+$ cell subsets in CKD (B) and lymphatics and $CD4^+$ cell subsets in non-alloimmune graft injury (C). Each node represents a putative ligand or receptor, and each line represents an interaction, with stimulatory interactions coloured in red and inhibitory interactions coloured in blue. The size of the node represents the proportion of cells expressing the ligand or receptor, and the darkness of the line represents the strength of the CellPhoneDB interaction.

SUPPLEMENTARY VIDEO LEGENDS

Video S1. 3D confocal microscopy of the lymphatic vasculature in healthy human kidney

Three-dimensional reconstruction of healthy human kidney tissue stained with the D2-40 monoclonal antibody (green, PDPN) to label lymphatic vessels. Autofluorescent structures are shown in blue. Acquired using two-photon microscopy, the video begins with rotation, followed by high-magnification panning through the cortex, illustrating the architecture of the lymphatic plexus and its blind-ended capillary initiation.

Video S2. Arterial association and lymphatic vessel hierarchy in healthy human kidney

3D reconstruction of healthy human kidney tissue stained for PDPN (green) to label lymphatics. Autofluorescence (blue) highlights large arteries, revealing their close anatomical proximity to lymphatic vessels. The video rotates and pans to show lymphatic capillaries branching from arterial regions into the cortex, ending in blind tips.

Video S3. Cortical lymphatic initiation adjacent to proximal tubules in human kidney

3D reconstruction of healthy human kidney tissue co-stained for PDPN (green) and *Lotus tetragonolobus* lectin (LTL, orange), marking proximal tubules. The video alternates between channels and pans through at high magnification, highlighting the emergence of blind-ended lymphatic capillaries adjacent to proximal tubular epithelium.

Video S4. Lymphatic initiation near distal and connecting tubules in human kidney

3D reconstruction showing PDPN (green) for lymphatics and CALB1 (yellow) marking distal/connecting tubules in healthy human kidney. The video pans and rotates, demonstrating blind-ended lymphatic capillaries closely associated with distal nephron segments in the cortex.

Video S5. Spatial organization of lymphatics and collecting ducts in healthy kidney
3D reconstruction of healthy human kidney tissue stained with PDPN (green) for lymphatics and *Dolichos biflorus* agglutinin (DBA, red) for collecting ducts. The rotating image shows the anatomical arrangement between collecting ducts and surrounding lymphatic vessels.

Video S6. Lymphatic expansion in chronic kidney transplant rejection
3D reconstruction of kidney allograft tissue undergoing chronic rejection. PDPN (green) labels lymphatics, DBA (red) labels collecting ducts. The image rotates to show the pronounced expansion of lymphatics compared to healthy kidney (see Video S5), and their spatial relationship to collecting ducts.

Video S7. Junctional architecture of endothelial cells in human kidney
3D reconstruction of healthy human kidney tissue stained with CDH5 (red), highlighting endothelial cell-cell junctions of both blood and lymphatic vasculature. The video pans and zooms to display the junctional organization within the intact cortical microvasculature.

Video S8. Lymphatic–tertiary lymphoid structure interactions in transplant rejection
3D reconstruction of chronically rejecting human kidney transplant tissue stained with PDPN (green, lymphatics), CD21 (blue, follicular dendritic cells), and PNAd (high endothelial venules). The video rotates, zooms, and pans to show tertiary lymphoid structures (TLS) interconnected by lymphatic vessels.

Video S9. Lymphatic-lymphocyte spatial relationships in chronic transplant rejection
3D reconstruction of kidney allograft tissue stained with PDPN (green), CD4 (red), and CD20 (grey), marking lymphatics, T cells, and B cells, respectively. The video introduces each channel, then zooms and pans to demonstrate lymphocyte proximity to lymphatic vessels. Toward the end, extraluminal lymphocytes are subtracted, revealing cells located within lymphatic lumens.

Video S10. HLA-DR-expressing lymphatics exhibit adjacent T cell accumulation in chronic transplant rejection

3D reconstruction of kidney allograft tissue stained for PDPN (green), HLA-DR (red), and CD3 (grey). The video zooms in on a lymphatic vessel, cropping surrounding tissue to focus on HLA-DR$^+$ regions and adjacent CD3$^+$ T cells. 3D renderings reveal close spatial relationships between CD3$^+$ T cells and HLA-DR$^+$ regions of lymphatics.

Organ-specific features of human kidney lymphatics are disrupted in chronic transplant rejection

Daniyal J Jafree[1,2,3], Benjamin J Stewart[4,5], Karen L Price[1,2], Maria Kolatsi-Joannou[1,2], Camille Laroche[1,2], Barian Mohidin[1,2], Benjamin Davis[6], Hannah Mitchell[7], Lauren G Russell[1,2], Lucía Marinas del Rey[2,8,9], Chun Jing Wang[9], William J Mason[1,2], Byung Il Lee[10], Lauren Heptinstall[2,11], Ayshwarya Subramanian[12], Gideon Pomeranz[1,2], Dale Moulding[1], Laura Wilson[1,2], Tahmina Wickenden[1,2], Saif N Malik[1,2], Natalie Holroyd[13], Claire L Walsh[13], Jennifer C Chandler[1,2], Kevin X Cao[1,2], Paul JD Winyard[1,2], Adrian S Woolf[14], Marc Aurel Busche[10], Simon Walker-Samuel[13], Lucy SK Walker[9], Tessa Crompton[15], Peter J Scambler[1], Reza Motallebzadeh[2,8,9], Menna R Clatworthy[4,5]*, David A Long[1,2]*

[1]Developmental Biology & Cancer Research & Teaching Department, UCL Great Ormond Street Institute of Child Health, UCL, London, UK; [2]UCL Centre for Kidney & Bladder Health, UCL, London, UK; [3]UCL MB/PhD Programme, UCL, London, UK; [4]Molecular Immunity Unit, University of Cambridge, Cambridge, UK; [5]Wellcome Sanger Institute, Hinxton, Cambridge, UK; [6]Central Laser Facility, Science and Technologies Facilities Council, UK Research and Innovation, Didcot, Oxfordshire, UK; [7]Mathematical Sciences Research Centre, Queen's University Belfast, Belfast, UK; [8]Research Department of Surgical Biotechnology, Division of Surgery and Interventional Science, UCL, London, UK ; [9]UCL Institute of Immunity and Transplantation, UCL, London, UK; [10]UK Dementia Research Institute at UCL, London, UK; [11]UCL Department of Pathology, Royal Free Hospital, London, UK; [12]Department of Molecular Biology and Genetics, College of Arts and Science, Cornell University, Ithaca, NY, USA; [13]UCL Centre for Advanced Biomedical Imaging, London, UK; [14]School of Biological Sciences, Faculty of Biology Medicine and Health, University of Manchester, Manchester, United Kingdom; [15]Infection, Immunity and Inflammation Research and Teaching Department, UCL Great Ormond Street Institute of Child Health, UCL, London, UK;

***: Corresponding authors**

Professor David A Long
Professor in Paediatric Nephrology & Wellcome Trust Investigator in Science,
Developmental Biology and Cancer Research & Teaching Department,
UCL Great Ormond Street Institute of Child Health
30 Guilford Street, London, WC1N 1EH, UK
Tel.: +44(0)2079052615, Email: d.long@ucl.ac.uk

Professor Menna R Clatworthy
Molecular Immunity Unit,
University of Cambridge Department of Medicine,
MRC Laboratory of Molecular Biology,
Cambridge Biomedical Campus,
Francis Crick Avenue, Cambridge. CB2 0QH, UK
Tel.: +44(0)1223267279, Email: mrc38@cam.ac.uk

Supplemental Materials and Methods

Sex as a biological variable

Due to the exploratory nature of 3D imaging and single-cell RNA sequencing performed in this study, and the limited kidneys available for 3D imaging analysis, sex was not considered as a biological variable.

Three-dimensional imaging and analysis of human kidney lymphatics

Acquisition, fixation and storage of human tissue for three-dimensional imaging

Human adult kidney tissue was derived from four deceased patients who had opted in for organ donation and undergone multi-organ procurement, but for whom the kidneys had ultimately been declined for implantation by recipient transplant centres. Kidneys were retrieved by a UK National Organ Retrieval Services teams. Following *in situ* flushing of the abdominal organs with University of Wisconsin (UW) solution, the kidneys were removed and stored in UW at 4°C. Kidney allograft samples were obtained from three patients at Royal Free London NHS Trust undergoing nephrectomy for graft intolerance syndrome (n = 2) or graft malignancy (n = 1). All explants were performed by the transplant surgical team. Prior to acquisition, all patients were confirmed negative for COVID-19 by means of a qPCR test. After explant, pseudo-anonymised human adult kidney tissues were incubated overnight in Belzer University of Washington Cold Storage Solution (Bridge to Life Europe, London, UK) at 4°C. Prior to fixation, human adult kidney was manually dissected into ~3mm full-thickness sub-regions containing cortex and outer medulla. These tissues were then incubated in 4% (w/v) paraformaldehyde (PFA, Sigma Aldrich), made up in 1 X phosphate buffered saline (PBS), at 4° C overnight. After fixation, all biological tissues were washed and stored in 1 X PBS with 0.02% (w/v) sodium azide to prevent contamination. Randomly selected pieces of human adult kidney were transferred to and stored in 70% ethanol for histology.

Wholemount immunofluorescence

A modified version of the SHANEL protocol (1) was implemented for wholemount immunolabelling of kidney tissues. Unless otherwise stated, steps were performed at room temperature, and reagents purchased from Sigma Aldrich. Tissues were dehydrated in a methanol series (50, 70%) in double distilled (dd)H2O, for one hour per step, before bleaching in absolute methanol with 5% (v/v) of 30% hydrogen peroxide solution overnight at 4°C. Thereafter, tissues were rehydrated in the methanol series, followed by incubation in 1 x PBS for one hour. Overnight incubation of tissues was performed in a 0.5 M solution of acetic acid at 4°C, followed by five hours of incubation at 4°C with 4 M guanidine hydrochloride, 0.05 M sodium acetate and 2% (v/v) Triton X-100 made up in PBS. Tissues were then permeabilised with 5% (w/v) solution of 3-((3-cholamidopropyl) dimethylammonio)-1-propanesulfonate (CHAPS) made up in ddH2O overnight. Then tissues were incubated for one day in blocking solution, comprising 1 x PBS with 0.2% Triton X-100, 5% (v/v) donkey or goat serum, 5% (v/v) pooled human plasma (Biowest, Nuaillé, France) and 10% (v/v) dimethyl sulfoxide (DMSO) before incubation in antibody solution (1 x PBS with 0.2% (v/v) Tween-20, 0.1% (v/v) of a 10mg/ml heparin solution in ddH2O, 0.1% (w/v) saponin, 2.5% donkey or goat serum, 2.5% pooled human plasma with primary antibodies at the appropriate concentration at 4°C. Blocking and antibody solutions were further supplemented with 1:150 Human TruStain FcX™ Fc Receptor Blocking Solution (BioLegend, London, UK), to reduce non-specific binding. Primary antibodies were incubated for 3-4 days, before replenishing the antibody solution and re-incubation for 3-4 days. Subsequently, tissues were washed in 1 x PBS with 0.2% Tween-20 four times for 1 hour per wash, before incubation in antibody solution with secondary antibodies at 1:200 at 4°C for four days. Tissues were then washed again in 1 x PBS with 0.2% Tween-20 four times for 1 hour each and stored until dehydration and clearing.

Primary antibodies, lectins and secondary antibodies

In order of appearance in the manuscript, the following primary antibodies or lectins were used in 1.5ml incubations at the indicated concentrations: mouse anti-PDPN monoclonal (clone:

D2-40, 1:100, M3619, Aligent), rabbit anti-PROX1 polyclonal (1:200, ABN278, Merck), goat anti-LYVE1 polyclonal (1:100, AF2089, R&D Systems), fluorescein-conjugated LTL (1:50, Vector Laboratories), rabbit anti-UMOD monoclonal (clone: EPR20071, 1:100, ab207170, Abcam), fluorescein-conjugated UAE-I (1:50, Vector Laboratories), rabbit anti-LRP2 polyclonal (1:50, ab76969, Abcam), rabbit anti-CALB1 monoclonal (clone: EP3478, 1:100, ab108404, Abcam), rhodamine-conjugated DBA (1:50, Vector Laboratories), mouse anti-CDH1 monoclonal (clone: HECD-1, 1:50, ab1416, Abcam), mouse anti-PECAM1 monoclonal (clone: JC70A, 1:50, M0823, Dako), mouse anti-CD68 monoclonal (clone: KP1, 1:100, ab955, Abcam), rabbit anti-αSMA polyclonal (1:50, ab5694, Abcam), rabbit anti-HLA-DR monoclonal (clone: EPR3692, 1:100, ab92511, Abcam), rabbit anti-C4d polyclonal (1:100, 0300-0230, Bio-Rad), rabbit anti-CD4 monoclonal (clone: EPR6855, 1:100, ab133616, Abcam), goat anti-CD20 polyclonal (1:100, ab194970, Abcam), goat anti-PVR polyclonal (1:100, AF2530, R&D Systems), rabbit anti-CD21 monoclonal (clone: EPR3093, 1:200, ab75985, Abcam), rat anti-PNAd monoclonal (clone: MECA-79, 1:100, MABF2050, Sigma), goat anti-CDH5 polyclonal (1:50, AF938, R&D Systems). All secondary antibodies were purchased from ThermoFisher Scientific, were conjugated to AlexaFluor fluorophores (488, 546, 568, 633 or 647) and were used at a concentration of 1:200 of the original secondary antibody stock. Controls for each panel involved omission of the primary antibody and including the secondary antibody only.

Solvent-based optical clearing

Tissues were dehydrated in a methanol series (50%, 70%, 100%) for 1 hour per step. BABB (benzyl alcohol and benzyl benzoate in a 1:2 ratio), was used for clearing, with all solutions containing BABB kept in glass scintillation vials (VWR International, Lutterworth, UK). Clearing was performed in glass scintillation vials, first using BABB:methanol in a 1:1 ratio, and thereafter BABB alone, until samples equilibrated and achieved transparency.

Confocal microscopy

We took advantage of the z-depth achievable by upright confocal microscopy whilst protecting the microscope objectives. All tissues were placed between a large coverslip and cover glass, supported by a O-Ring (Polymax Ltd, Bordon, UK) made from BABB-resistant rubber, as described previously (2). Confocal images were acquired on an LSM880 upright confocal microscope (Carl Zeiss Ltd.), with a 2.5x/numerical aperture (NA) 0.085 Pan-Neofluar Dry objective (working distance; WD = 8,800 µm) for low-resolution imaging, and 10×/NA 0.5 W-Plan Apochromat water dipping objective (working distance; WD = 3,700 µm) for high-resolution imaging. Gallium arsenide phosphide (GaAsP) internal and external detectors were used for high sensitivity. To obtain higher resolution imaging, an Airyscan setting (3), consisting of a 32-channel (GaAsP) photomultiplier tube area detector.

Lightsheet fluorescence microscopy

3D imaging of cleared tissues was performed using a custom-built mesoscale selective plane illumination microscope (mesoSPIM) (4). The cleared tissue was secured in a 3D-printed holder and immersed in BABB solutions inside a quartz cuvette (40 x 40 x 100 mm). Fluorescence images were acquired with an Olympus MVX-10 macroscope at 1x magnification, resulting in a voxel size of 6.55 x 6.55 x 5 μm^3. PDPN fluorescence signals were obtained using 638 nm laser excitation and 633nm long-pass optical filtering of emitted light, while autofluorescence was captured using 488 nm laser excitation and a 520/35 nm bandpass emission filter. Lightsheet illumination from both sides of the cuvette was carefully aligned after the sample was positioned at the centre of the macroscope's field of view and delivered simultaneously to capture a single *z*-stack image.

Post-acquisition image processing

All images were then exported to FIJI (NIH, Bethesda, US). Confocal image stacks were separated into individual fluorescence channels, and the Despeckle and Sharpen tools were used to reduce non-specific background fluorescence. Where maximum intensity *z*-

projections or optical *z*-sections were required, scale bars were applied and images and exported as TIFF files.

Image visualisation and binarization of three-dimensional imaging data

Visualisation of 3D reconstructions were performed by importing confocal images to the commercial software, Imaris (v8.2, Bitplane). The Isosurface Rendering tool in Imaris allows the extraction of surfaces based on fluorescence intensity. This was used to generate segmented images fluorescence masks to visualise expression patterns, or to generate binarized outputs for extraction of vessel branching metrics. LSFM data was imported into Amira (v2020.2, Fisher Scientific) and the vasculature segmented using intensity thresholding and region growing using the Magic Wand tool to generate a binarized network.

Extraction of vessel branching metrics from three-dimensional imaging data

Segmented and binarized confocal and LSFM images were imported as TIFF image stacks into Amira. The Filament Editor tool was used in Amira to generate spatial statistical parameters including vessel branch number, lengths, diameter and volumes from each segmented lymphatic plexus. The resulting values were exported these as CSV files.

Assessment of lymphatic cell-cell junctional architecture

Volumes of interest of kidney tissues co-labelled with PDPN and CDH5 were segmented in IMARIS, generating a mask of PDPN signal to discriminate lymphatic-derived $CDH5^+$ signal from non-lymphatic-derived $CDH5^+$ signal, the latter corresponding to the blood vasculature. The volume of the lymphatic network from each image was determined, and the number of segmented and rendered discontinuous $CDH5^+$ structures was counted. As the lymphatic network in rejecting allografts was significantly larger, each value of discontinuous $CDH5^+$ junctions was volume normalised according to the volume of interest.

Spatial statistical analysis of lymphatic-lymphocyte relationships

Lymphatic 3D-skeletons were extracted from binarised confocal stacks using the BoneJ Skeletonise3d function in FIJI (5). $CD4^+$ T cell and $CD20^+$ B cell counts, centroids and areas were obtained using 3d-objectcounter with no further pre-processing (6). The mean distance of each cell from the nearest point of the lymphatic network (*d*) was calculated using the cross-product 3D point-line distance:

$$d = \frac{|(x_0 - x_1) \times (x_0 - x_2)|}{|(x_2 - x_1)|}$$

where x_1 and x_2 are the two closest adjacent nodes from the lymphatic 3D skeleton; found by minimizing cross-nearest neighbor distances, and x_0 is the centroid of the cell of interest. To evaluate whether the cell distances were different from what would be expected by chance, within each region of interest, the $CD4^+$ T cell and $CD20^+$ B cell populations were randomly redistributed under complete spatial randomness for twenty simulations. A comparison was then made as to whether the measured mean cell-lymphatic distances fell within the 95% confidence intervals obtained through the simulations under complete spatial randomness.

Single-cell transcriptomic analysis of human kidney lymphatics

Acquisition of material for single-cell transcriptomics and generation of a human kidney cell atlas

The scRNA-seq dataset from this study consisted of previously published data and five new samples. For the five new samples, kidney allografts undergoing graft nephrectomies were collected at the time of surgery. Tissue was digested and processed to a single cell suspension as described previously (7). Cells were counted using a haemocytometer and cell concentrations adjusted using dilution in RPMI Medium (Sigma) to a concentration of 1000 cells/µL. Cells were loaded according to the protocol of the 10X Chromium single cell 5'v2 kit to capture 10,000 cells per channel. Libraries were prepared according to manufacturer

instructions and sequencing was performed on an Ilumina Novaseq instrument with read lengths specified by 10X genomics. Sequencing data were mapped against GRCh38-3.0.0 using 10X Cellranger V6.0.2. These data were merged with previously published scRNA-seq data of the human kidney, including samples from non-tumorous regions of tumour nephrectomies with or without CKD (7, 8), live allograft biopsies with or without antibody-mediated rejection (9) and human kidney data from the Kidney Precision Medicine project (10). Data were merged using anndata (0.8.0), using a nuisance gene mask as previously described (7) to minimise the effect of technical noise on batch integration. Thereafter, the data were split into healthy and diseased datasets using publicly available metadata. Highly variable genes were calculated using scanpy (1.8.2) setting *n_top_genes* = 1500, *batch_key* = 'dataset', and flavour = '*seurat_v3'* and using raw counts. On the basis of these variable genes, we constructed a variational autoencoder model using scArches (11), using both dataset and the donor identity as categorical covariate keys, and a dissociation stress score calculated from aggregate dissociation-induced gene expression (12) as a continuous covariate. A single cell variational inference (scVI) model was trained using n_layers = 2, and n_latent = 15. We then used the latent space computed from this model as input to scanpy's neighbourhood graph calculation and UMAP computation functions. Data were clustered using Leiden clustering and annotated on the basis of marker genes. Immune cell annotations were further supported by prediction from a logistic regression model with the CellTypist python package. The data was then converted using seurat-disk before further analysis using the Seurat package in R. Unless otherwise stated, all downstream steps were performed in Seurat.

Analysis of kidney lymphatic transcriptional heterogeneity

To capture heterogeneity of lymphatic cells within the human kidney, the cell cluster corresponding to lymphatics was extracted from the control kidney dataset, and a raw count matrix was generated from all 295 lymphatic cells. We combined this with data from a recent study utilising scRNA-seq to examine nine human control kidneys after tumour nephrectomies

(13). From the latter dataset, a cluster of 157 lymphatic cells was identified and the raw count matrix merged with our dataset, giving a total of 452 lymphatic cells. A subset of newly generated count matrix was created to only include genes detected in both datasets, resulting in 15,316 genes. The data was pre-processed using the Seurat workflow including normalisation, scaling by all genes and principal component (PC) analysis. Integration of the two lymphatic cell datasets was achieved using the Harmony package, integrating by the study from which the cells were sourced. The *FindNeighbors, FindClusters* and RunUMAP algorithms were then computed, using 7 PCs, a resolution of 0.4 and utilising the embeddings generated by Harmony integration. Differential expression analysis was performed as below to discriminate subcluster-specific markers from the two transcriptionally distinct clusters detected.

Creation of a human organ lymphatic atlas

Count matrices of publically available datasets from the Human Cell Atlas, including skin (14), breast (15), heart (16, 17), lung (18) and small or large intestines (19), were downloaded. Lymphatic endothelium was identified and isolated from the metadata of each dataset and individually curated for the expression of *PROX1* and *PDPN*. We generated a count matrix which included each of these datasets, and the 452 lymphatic cells from control kidneys computationally isolated as aforementioned. Genes that not detected across all datasets or that were not represented in all count matrices were deleted to avoid artefactual clustering. The analysis workflow described as above, including Harmony integration, was then used, performing integration by individual donor. Predicted transcription factor activity was assessed using the SCENIC package in R.

Assessment of kidney lymphatic-enriched candidates in human kidney diseases

To assess the expression of *DNASE1L3* and *MDK* across a range of human kidney diseases, the NephroSeq database was used. We searched for RNAseq or microarray expression datasets containing these transcripts, only including datasets that assessed tubulointerstitial

gene expression. The datasets were compiled and $\log_2$ expression values were extracted and plotting using Violin plots.

Differential expression analysis

The *FindAllMarkers* function was used for differential expression analysis of scRNAseq data. Wilcoxon Rank Sum tests were used to assess statistically significant (adjusted p value ≤ 0.05) between average log fold change values of expression. Selected differentially expressed genes were visually represented using the *VlnPlot* function, the *DoHeatMap* function or using the EnhancedVolcano (https://github.com/kevinblighe/EnhancedVolcano) package. For NephroSeq data, $\log_2$ expression values were assessed using ANOVA tests with Tukey post-hoc correction for individual comparisons.

Gene ontology analysis

Gene ontology (GO) analysis was performed using the PANTHER tool for gene classification. Lists of differentially expressed genes, were exported and input into the PANTHER web tool (v16.0, http://www.pantherdb.org), using statistical overrepresentation tests to group genes using the GO biological processes complete database. Fisher's Exact tests were used to assess for statistical enrichment of genes for selected GO terms, and a false discovery rate (FDR) p ≤ 0.05 was considered significant.

CellPhoneDB

To infer putative cell-cell interactions in single-cell RNA sequencing data, the CellPhoneDB resource (20) was used. Using normalised count and metadata files obtained from Seurat, CellPhoneDB was called by running appropriate commands, obtained from https://github.com/Teichlab/cellphonedb, in the command line through a Python virtual environment. The *statistical_analysis* method was used to assess predicted interactions, before functions in ktPlots (https://github.com/zktuong/ktplots) were used to generate custom dot plots or circle plots.

Lymphatic endothelial cell stimulation assays

Cell line and recombinant IFNγ treatment

Adult human dermal lymphatic endothelial cells (HDLECs; PromoCell GmbH) were cultured in Endothelial Cell Growth Medium MV2 (PromoCell) at 37 °C in a 5% CO_2 incubator. Media were refreshed twice weekly, and confluent cultures were passaged at a 1:3 ratio. cells (*n* = 2 independent lines) were seeded at a density of 1 x 10^5 in a 6-well plate, treated with either normal growth media (unstimulated group) or IFNγ (50ng/ml; Thermo Fisher Scientific,) for 24 hours, 48 hours and 72 hours.

Quantitative RT-PCR for LGALS9 expression by HDLECs

To assess *LGALS9* RNA expression, cells at each time point were harvested, lysed and total RNA extracted using the RNeasy Plus Mini Kit (Qiagen). cDNA was synthesised from 500 ng RNA using the iScript cDNA Synthesis Kit (Bio-Rad Laboratories). Quantitative real-time PCR was performed on a CFX96 Real-Time PCR System (Bio-Rad) using qPCRBIO SyGreen Mix Lo-ROX (PCR Biosystems Ltd). Expression of *LGALS9* was normalised to the housekeeping gene *HPRT*, and fold-change was calculated using the $2^{-\Delta\Delta CT}$ method. Results were standardised to the mean expression in untreated controls (set to 1). The experiment was repeated three times and all assays performed in duplicate. The presented data shows the mean fold-change in gene expression measured in three independent cell lines. Primer sequences are available upon request.

Assessment and quantification of LGALS9 secretion by HDLECs

To assess LGALS9 protein secretion, conditioned media from HDLECs at each timepoint were collected and centrifuged to remove cell debris. Galectin-9 levels were quantified by ELISA

(DGAL90, R&D Systems) according to the manufacturer's instructions. Optical density was measured at 450 nm, and protein concentration was calculated using a standard curve derived from serial dilutions of recombinant LGALS9. The experiment was repeated three times and all assays performed in duplicate. Results were expressed relative to unstimulated wells, which were standardised to a value of 1.

Statistical analysis, data presentation and availability

Sample size estimation

In prior work examining the 3D architecture of lymphatic vessels in lymphangiomatous skin biopsies, conclusions were drawn based on the evaluation of three samples within the control group (21), and so a minimum of three patients per group were used to draw conclusions. For scRNA-seq, the number of samples and cells to be analysed was limited by the size of the dataset. The specific number of replicates used for each experiment and the number of regions images are indicated in the figure legends,

Reproducibility and data presentation

Descriptive conclusions are drawn based on a minimum of four imaging volumes of interest, each taken from samples from at least two different human kidneys. All confocal and brightfield images were exported and saved as TIFF files. Where brightness or contrast were adjusted, this was applied uniformly across all conditions within the same figure, and details are stated in figure legends. Cell culture experiments were performed in biological triplicates, with a minimum of two technical replicates for each assay. Graphs were generated in GraphPad PRISM and saved as TIFF format. Visualisations from scRNA-seq analysis were performed in RStudio and PNG screenshots were taken and saved. Figures were compiled in Microsoft PowerPoint (Microsoft, Redmond, US) and saved as PDF format.

Statistics

Except for scRNA-seq analysis and lymphatic-lymphocyte spatial relationships, all remaining statistical comparisons were performed using GraphPad PRISM. A two-tailed *p* value of less than 0.05 was considered statistically significant. For continuous data, Shapiro-Wilk tests were used to assess normality of distribution and Brown-Forsythe tests were used equality of variance. Where normal distribution and equality of variances were satisfied, data is presented as mean ± standard deviation. When graphed, error bars were used to represent the standard error of the mean. Student's *t*-test was used to compare two groups and ANOVA was used to compare more than two groups, applying post-hoc Bonferroni tests to provide adjusted *p* values for multiple comparisons. Statistics for scRNA-seq analysis were performed in RStudio and are as detailed above.

Study approval

For reference human kidney tissue, consent for the use of the organs for research was obtained from the donor family by Specialist Nurses in Organ Donation before organ retrieval and were then offered for research by NHS Blood & Transplant (NHSBT) if they were found to be unsuitable for transplantation by the surgical team. Ethical approval was granted by the National Research Ethics Committee in the UK (21/WA/0388) and was approved by The Royal Free London NHS Foundation Trust-UCL Biobank Ethical Review Committee (RFL B-ERC; NC.2018.010; IRAS 208955). Ethical approval to obtain explants with chronic rejection was covered by a prior agreement (NC.2018.007, UCL Biobank Ethical Review Committee, Royal Free London NHS Foundation Trust, B-ERC-RF). Human kidney tissues for scRNA-seq were explanted and processed at Cambridge University Hospitals NHS Foundation Trust under ethical approval (REC 16/EE/0014). Ethical approval for publicly available data for human lymphatic cells acquired from scRNA-seq experiments of kidney (22), skin (14), breast (15), heart (16, 17), lung (18) and intestines (19) are detailed in the original studies.

Data availability

Raw sequencing data for the five new human kidney scRNAseq samples have been made publicly accessible via the European Genome-phenome Archive (Accession number: EGAD00001015631). The annotated and processed Seurat objects and h5ad files are publicly available: https://doi.org/10.5281/zenodo.7566982. The code for analysis of the human kidney scRNA-seq atlas is available at https://github.com/daniyal-jafree1995/. The imaging data used is available from the lead contact upon request. All raw data used to plot graphs, except for scRNA-seq analyses, are provided within the Supporting Data Values document.

REFERENCES

1. Zhao S et al. Cellular and molecular probing of intact human organs.. *Cell* 2020;180(4):796–812.e19.
2. Jafree DJ, Long DA, Scambler PJ, Moulding D. Tissue Clearing and Deep Imaging of the Kidney Using Confocal and Two-Photon Microscopy.. *Methods Mol. Biol.* 2020;2067:103–126.
3. Huff J. The Airyscan detector from ZEISS: confocal imaging with improved signal-to-noise ratio and super-resolution. *Nat. Methods* 2015;12(12):i–ii.
4. Voigt FF et al. The mesoSPIM initiative: open-source light-sheet microscopes for imaging cleared tissue.. *Nat. Methods* 2019;16(11):1105–1108.
5. Lee TC, Kashyap RL, Chu CN. Building Skeleton Models via 3-D Medial Surface Axis Thinning Algorithms. *CVGIP: Graphical Models and Image Processing* 1994;56(6):462–478.
6. Bolte S, Cordelières FP. A guided tour into subcellular colocalization analysis in light microscopy.. *J. Microsc.* 2006;224(Pt 3):213–232.
7. Stewart BJ et al. Spatiotemporal immune zonation of the human kidney.. *Science* 2019;365(6460):1461–1466.
8. Kuppe C et al. Decoding myofibroblast origins in human kidney fibrosis.. *Nature* 2021;589(7841):281–286.
9. Malone AF et al. Harnessing expressed single nucleotide variation and single cell RNA sequencing to define immune cell chimerism in the rejecting kidney transplant.. *J. Am. Soc. Nephrol.* 2020;31(9):1977–1986.
10. Menon R et al. Single cell transcriptomics identifies focal segmental glomerulosclerosis remission endothelial biomarker.. *JCI Insight* 2020;5(6). doi:10.1172/jci.insight.133267
11. Lotfollahi M et al. Mapping single-cell data to reference atlases by transfer learning.. *Nat. Biotechnol.* 2022;40(1):121–130.
12. van den Brink SC et al. Single-cell sequencing reveals dissociation-induced gene expression in tissue subpopulations.. *Nat. Methods* 2017;14(10):935–936.
13. Subramanian A et al. Obesity-instructed *TREM2* macrophages identified by comparative analysis of diabetic mouse and human kidney at single cell resolution. *BioRxiv* [published online ahead of print: May 30, 2021]; doi:10.1101/2021.05.30.446342
14. Reynolds G et al. Developmental cell programs are co-opted in inflammatory skin disease.. *Science* 2021;371(6527). doi:10.1126/science.aba6500
15. Kumar T et al. A spatially resolved single-cell genomic atlas of the adult human breast.. *Nature* 2023;620(7972):181–191.
16. Kanemaru K et al. Spatially resolved multiomics of human cardiac niches.. *Nature* 2023;619(7971):801–810.
17. Litviňuková M et al. Cells of the adult human heart.. *Nature* 2020;588(7838):466–472.
18. Sikkema L et al. An integrated cell atlas of the lung in health and disease.. *Nat. Med.* 2023;29(6):1563–1577.
19. Elmentaite R et al. Cells of the human intestinal tract mapped across space and time.. *Nature* 2021;597(7875):250–255.
20. Efremova M, Vento-Tormo M, Teichmann SA, Vento-Tormo R. CellPhoneDB: inferring cell-cell communication from combined expression of multi-subunit ligand-receptor complexes.. *Nat. Protoc.* 2020;15(4):1484–1506.
21. Hägerling R et al. VIPAR, a quantitative approach to 3D histopathology applied to lymphatic malformations.. *JCI Insight* 2017;2(16). doi:10.1172/jci.insight.93424
22. Subramanian A et al. Obesity-instructed *TREM2* macrophages identified by comparative analysis of diabetic mouse and human kidney at single cell resolution. *BioRxiv* [published online ahead of print: May 30, 2021]; doi:10.1101/2021.05.30.446342

ID	DONOR DEMOGRAPHICS				RENAL FUNCTION		ORGAN CHARACTERISTICS		
	Age	Sex	Ethnicity	Cause of death	Serum creatinine	eGFR	Type of organ	Reason for decline	Histological report
**NK1**	68	M	Cauc.	Intraparenchymal haemorrhage	74	90	DBD	Cancerous lesions on contralateral kidney	Chronic damage <2%; 7/115 obsolete glomeruli; mild tubular blebbing; mild fibro-intimal proliferation of vessels
**NK2**	61	M	Cauc.	Acute cerebral infarction	94	75	DCD	Severe aortic atherosclerosis	Chronic damage <10%; 5/60 obsolete glomeruli; occasional tubular flattening, blebbing and vacuolation; mild fibro-intimal proliferation of vessels
**NK3**	57	M	Cauc.	Hypoxic brain injury	60	90	DCD	Severe aortic atherosclerosis	Chronic damage <10%; mild lymphocytic infiltrate; 7/80 obsolete glomeruli. occasional occasional tubular flattening, blebbing and vacuolation; mild focal fibro-intimal proliferation of vessels
**NK4**	22	M	Cauc.	Traumatic head injury	89	90	DCD	Hydronephrosis and PUJ obstruction	Chronic damage ~0%; 0/69 obsolete glomeruli; mild tubular flattening; mild fibro-intimal proliferation of vessels

Table S1. Clinicopathological information for organ donors from which control tissues were derived for 3D imaging. Age in years, serum creatinine in μmol/L, estimated glomerular filtration rate (eGFR) in ml/min/1.73m^2. Cauc., Caucasian; DBD, donor after brainstem death; DCD, donor after cardiac death; PUJ, pelvic ureteric junction

ID	DONOR DEMOGRAPHICS			TRANSPLANT CHARACTERISTICS				REJECTION INFORMATION		
	Age	Sex	Ethnicit y	Year of transpla nt	Type of organ	HLA mismatches	Drugs received	Indications for explant	Donor-specific antibodies	Histological report
**CR 1**	41	M	Cauc.	2006	Cadaveric	HLA-A (2)<br>HLA-B (1)<br>HLA-DR (1)	MMF tacrolimus	Failing transplant and two cancerous lesions	Anti-HLA-DQ5 (10778 MFI)	IF/TA present; glomerulosclerosis; moderate fibro-intimal proliferation of vessels with lymphocyte invasion; hyaline arteriosclerosis; tubulitis; interstitial lymphoplasmacytic infiltrate. Features of both TCMR and ABMR.
**CR 2**	79	F	Asian	2007	Live	Unknown	MMF cyclosporine	Failing transplant and graft intolerance	Unknown	IF/TA present; ischaemic glomeruli; peritubular capillaritis, endarteritis and chronic vasculopathy; tubilitis; interstitial lymphoplasmacytic infiltrate. Features of both TCMR and ABMR.
**CR 3**	76	F	Asian	1995	Cadaveric	HLA-A (1)<br>HLA-B (1)<br>HLA-DR (0)	Cyclosporine	Failing transplant and pain over graft	Anti-HLA-A2 (MFI 19807) Anti-HLA-A31 (MFI 25896) Anti-HLA-B60 (MFI 24681)	IF/TA present; mostly obsolete glomeruli; severe fibro-intimal proliferation of vessels with endarteritis; interstitial lymphoplasmacytic infiltrate; calcification and simple cysts present. Features of both TCMR and ABMR present.

Table S2. Clinicopathological information for patients from whom transplant rejection tissues were derived for 3D imaging. Age in years. Cauc., Caucasian; DBD, donor after brainstem death; DCD, donor after cardiac death; HLA, human leukocyte antigen; MFI, mean fluorescence intensity; MMF, mycophenolate mofetil.
